# Supplementary material for: Epigenetic biomarkers predict macrovascular events in individuals with type 2 diabetes
Source: Cell Rep Med. 2025 Aug 7;6(8):102290. doi: 10.1016/j.xcrm.2025.102290 (PMC12432358; doi:10.1016/j.xcrm.2025.102290)
Supplement: Document S1. Figures S1–S4 [file mmc1.pdf]

Cell Reports Medicine, Volume 6

Supplemental information

Epigenetic biomarkers predict macrovascular events in individuals with type 2 diabetes

Sonia García-Calzón, Alice Maguolo, Fabian Eichelmann, Andreas Edsfeldt, Alexander Perfilyev, Marlena Maziarz, Axel Lindström, Jiangming Sun, Monta Briviba, Matthias B. Schulze, Janis Klovins, Emma Ahlqvist, Isabel Gonçalves, and Charlotte Ling

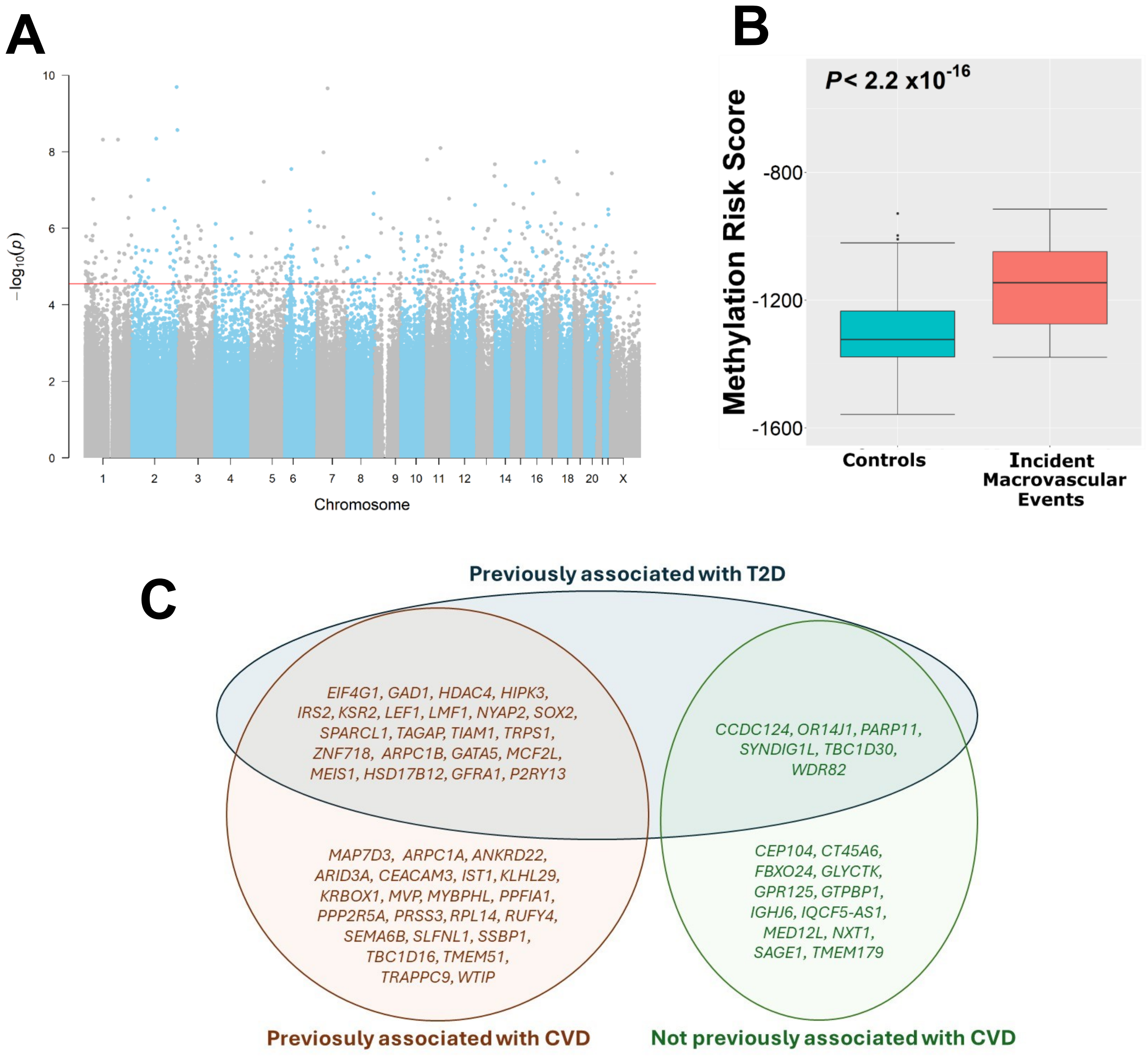

Figure S1. Blood DNA methylation is associated with incident macrovascular events (iME) in newly-diagnosed individuals with type 2 diabetes (T2D) and shows functional relevance in annotated genes, Related to Figures 2 and 6 (A) Manhattan plot showing the chromosomal distribution and significance levels of all sites analyzed (Illumina MethylationEPIC Beadchip array, 853,307 sites). The significance cut off (red line) was false discovery rate <5%. 461 sites were found significant($q<0.05$) in the *prospective cohort for macrovascular events in T2D,* after adjusting for age, gender, BMI and HbA1c. **(B)** A Methylation Risk Score (MRS), based on the 87 methylation sites presented in Table S2B, was different between individuals who developed macrovascular events and controls who did not develop macrovascular events after 7 years of follow-up in newly-diagnosed individuals with T2D. The boxplot shows significantly different MRS between controls (n=650) and incident macrovascular events (n=102) in the *prospective cohort for macrovascular events in T2D* (*P* for Mann-Whitney U test $< 2.2 \times 10^{-16}$). **(C)** Venn Diagram of the 64 genes annotated to the 87 methylation sites associated with iME in individuals with T2D included in the MRS showing an overview of the biological relevance assessment performed based on systematic PubMed and GWAS Catalog searches. The Venn diagram shows the genes that has been previously associated with cardiovascular phenotypes in the PubMed search “gene AND Vascular Disease OR Cardiovascular Disease”, “gene AND Myocardial Infarction”, “gene AND Stroke”, “gene AND Ischemic Heart Disease”, “gene AND Angina” (also shown in Figure 6A) OR with CVD-related traits based on GWAS Catalog search (also shown in Figure 6B) and the genes that have been previously associated with diabetes in PubMed search “gene” AND “diabetes” or GWAS Catalog search of “diabetes” trait. Overall, 46 of 64 genes (72%) have been previously associated with CVD and 18 (28%) have not; however, 6 of these 18 genes were associated with diabetes.

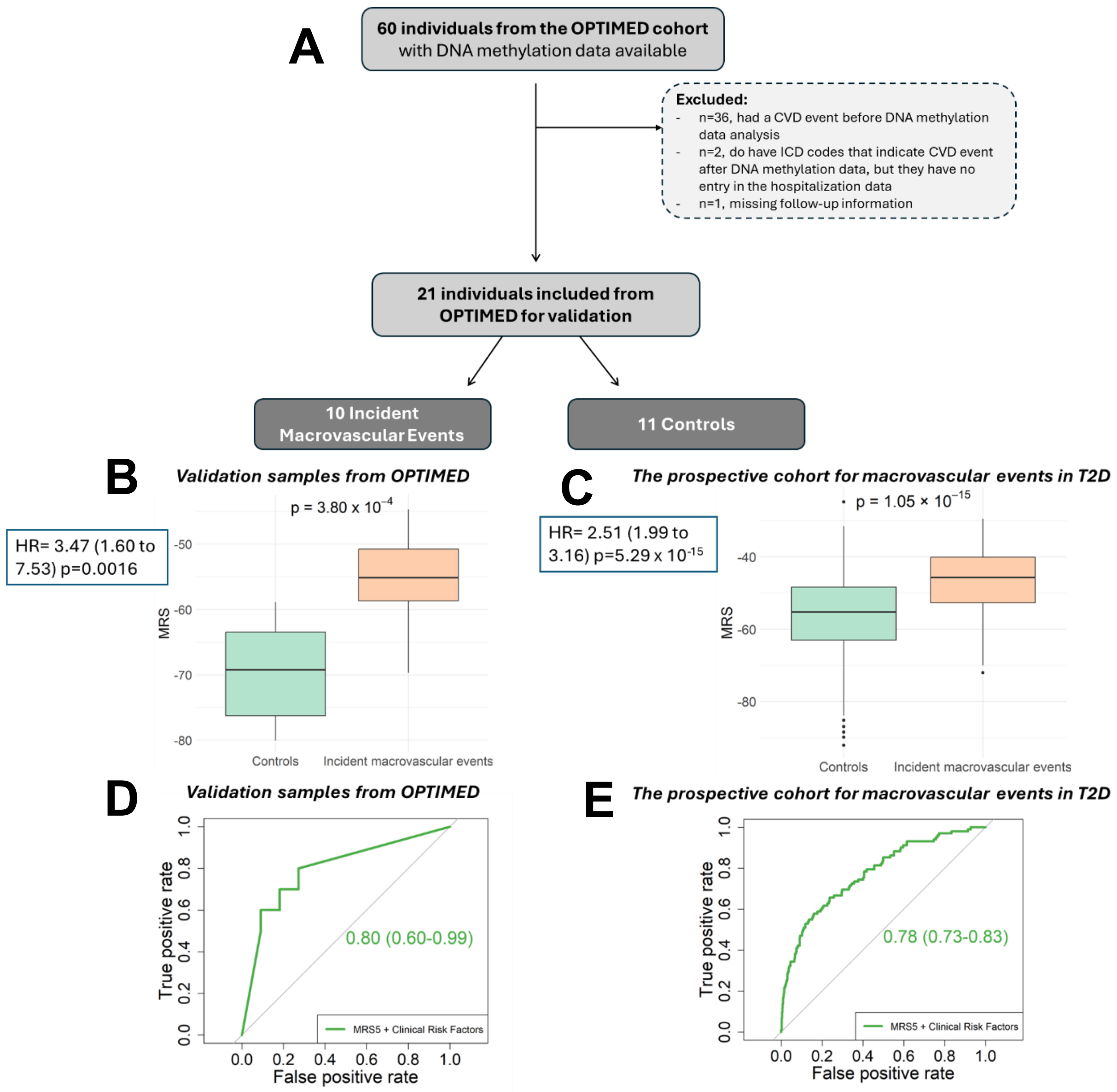

Figure S2. Validation of epigenetic markers associated with incident macrovascular events (iME) in newly-diagnosed individuals with type 2 diabetes (T2D) from the OPTIMED cohort. Related to Figure 1 and STAR methods. (A) Flowchart illustrating the selection and exclusion process of individuals from the OPTIMED cohort for validation, leading to the final analysis groups of iME and controls. **(B-E)** Methylation Risk Score (MRS_{5sites}), based on the five methylation sites presented in Table S5A, was different in blood taken from newly-diagnosed individuals with type 2 diabetes (T2D) between those who developed macrovascular events (iME) and controls who did not develop macrovascular events in (**B**) OPTIMED after 11 years of follow-up, and (**C**) *the prospective cohort for macrovascular events in T2D* after 7 years of follow-up. The boxplots show significantly different MRS_{5sites} between controls (n=11 and n=650, respectively) and iME (n=10 and n=102, respectively) in (**B**) OPTIMED (*P* for Mann-Whitney U test = =$3.8x10^{-4}$), and (**C**) *the prospective cohort for macrovascular events in T2D* (*P* for Mann-Whitney U test = $1.05x10^{-15}$). Individuals who developed macrovascular events showed a 2.5- to 3.5-fold increased risk compared to controls per 1 SD increase in the MRS_{5sites}, with a hazard ratio (HR) of 3.47 (p=0.016) in OPTIMED, and an HR of 2.51 (p=$5.29x10^{-15}$) in *the prospective cohort for macrovascular events in T2D*. The MRS_{5sites} predicts macrovascular events in newly-diagnosed individuals with T2D in (**C**) OPTIMED after 11 years of follow-up using cross-validation, and (**D**) *the prospective cohort for macrovascular events in T2D* after 7 years of follow-up using cross-validation. ROC curves were generated with macrovascular events as the outcome and the predicted risks of each individual obtained using cross-validation (k=3 and 5, respectively) for the MRS_{5sites}, for the clinical risk factors (age + gender + HbA1c + BMI + smoking (cg05575921 methylation) + diabetes medication + lipid-lowering medication + antihypertensives) and for the combination of both the MRS and clinical risk factors, separately.

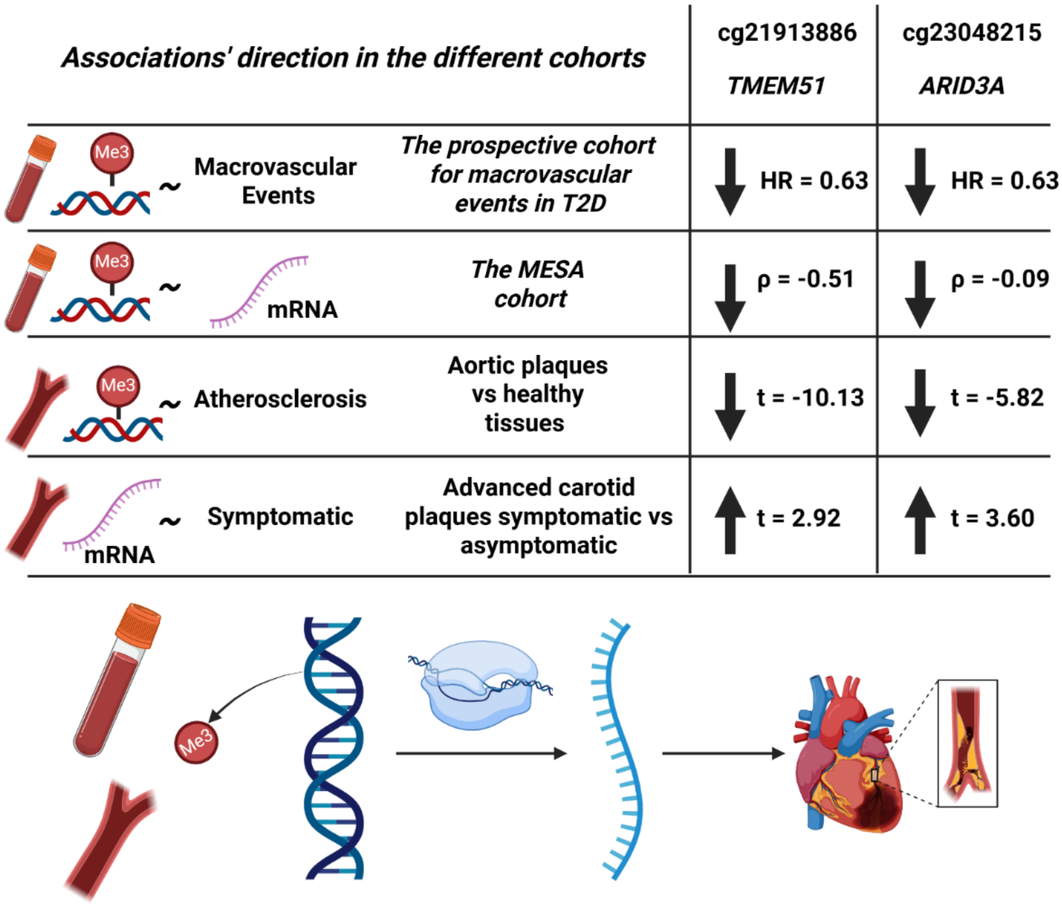

Figure S3. Concordance of associations' direction of DNA methylation and gene expression of the cg21913886 and cg23048215, respectively annotated to *TMEM51* and *ARID3A*, across different analyses and cohorts used in our study, **Related to Figure 6**. The figure shows that reduced DNA methylation in blood at these two CpG sites is associated with higher risk of macrovascular events in the *prospective cohort for macrovascular events in T2D* as well as with higher gene expression in the MESA cohort. Accordingly, we found that aortic plaques have reduced methylation at these sites compared to healthy aortic tissues and that symptomatic patients with advanced carotid plaques have higher expression of these genes compared to asymptomatic patients. Together, these analyses show that reduced methylation at cg21913886 and cg23048215 is associated with increased expression of *TMEM51* and *ARID3A*, respectively, that are associated with increased cardiovascular risk.

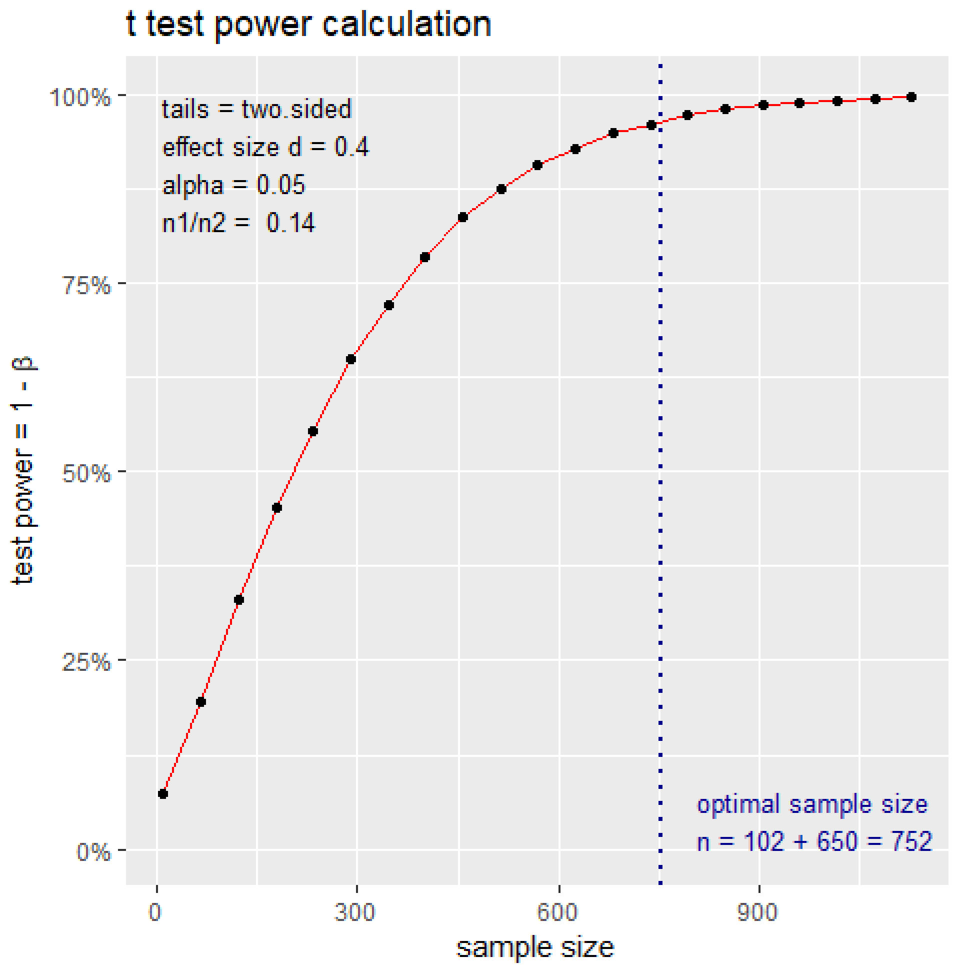

Figure S4. Statistical power calculations for the sample size in the *prospective cohort for macrovascular events in T2D*, Related to STAR Methods. A statistical power of 96% (α=0.05) was achieved to find 2% differences in methylation (SD=0.05) between controls (n=650) and incident macrovascular events (n=102) in the *prospective cohort for macrovascular events in T2D*. The pwr package in R was used to perform this plot.
