# Supplementary material for: Epigenetic biomarkers predict macrovascular events in individuals with type 2 diabetes
Source: Cell Rep Med. 2025 Aug 7;6(8):102290. doi: 10.1016/j.xcrm.2025.102290 (PMC12432358; doi:10.1016/j.xcrm.2025.102290)
Supplement: Document S2. Article plus supplemental information [file mmc9.pdf]

Cell Reports Medicine

Article

Epigenetic biomarkers predict macrovascular events in individuals with type 2 diabetes

Graphical abstract

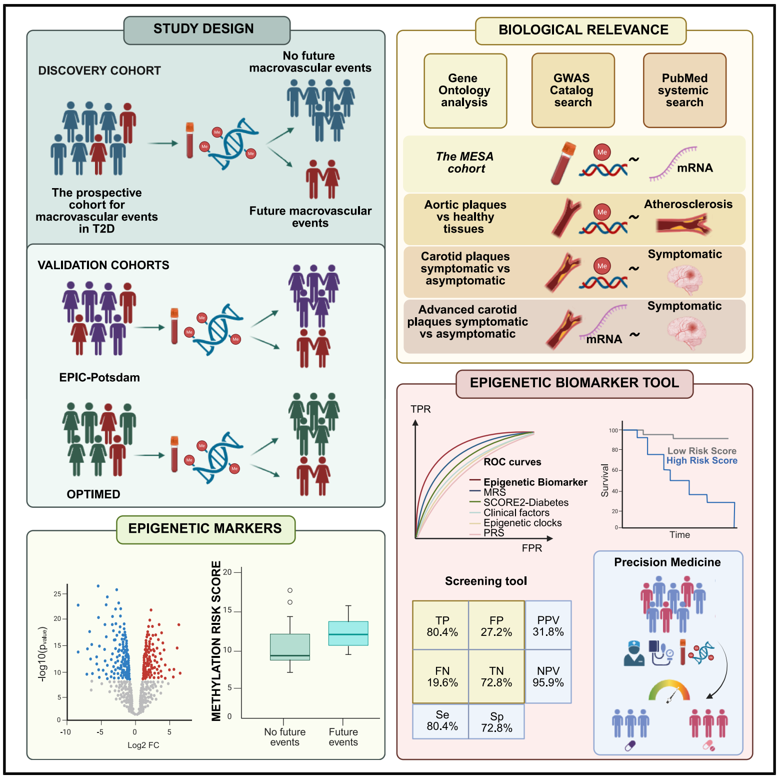

Highlights

- Blood-based epigenetic biomarker predicts macrovascular events at type 2 diabetes diagnosis
- The epigenetic biomarker tool outperforms established clinical cardiovascular risk scores
- DNA methylation markers also show biological relevance in human aortic and carotid plaques
- The epigenetic biomarker may support future precision medicine strategies in type 2 diabetes

Authors

Sonia García-Calzón, Alice Maguolo, Fabian Eichelmann, ..., Emma Ahlqvist, Isabel Gonçalves, Charlotte Ling

Correspondence

sgcalzon@unav.es (S.G.-C.),
charlotte.ling@med.lu.se (C.L.)

In brief

García-Calzón et al. identify a blood-based epigenetic biomarker tool that helps predict future cardiovascular disease, which individuals with type 2 diabetes are more likely to develop. Their findings support the use of epigenetic biomarkers to improve risk stratification and guide more personalized prevention strategies in diabetes care.

García-Calzón et al., 2025, Cell Reports Medicine 6, 102290
August 19, 2025 © 2025 The Author(s). Published by Elsevier Inc.
https://doi.org/10.1016/j.xcrm.2025.102290

Cell Reports Medicine

CellPress
OPEN ACCESS

Article

Epigenetic biomarkers predict macrovascular events in individuals with type 2 diabetes

Sonia García-Calzón,[1,2,3,*] Alice Maguolo,[1] Fabian Eichelmann,[4,5] Andreas Edsfeldt,[6,7,8] Alexander Perfilyev,[1] Marlena Maziarz,[9] Axel Lindström,[1] Jiangming Sun,[6] Monta Briviba,[10] Matthias B. Schulze,[4,5,11] Janis Klovins,[10,12] Emma Ahlqvist,[13] Isabel Gonçalves,[6,7] and Charlotte Ling[1,14,*]

[1]Epigenetics and Diabetes Unit, Department of Clinical Sciences Malmö, Lund University, Skåne University Hospital, 20502 Malmö, Sweden
[2]Department of Food Sciences and Physiology, Centre for Nutrition Research, IdiSNA, University of Navarra, 31008 Pamplona, Spain
[3]Centro de Investigación Biomédica en Red de Fisiopatología de la Obesidad y Nutrición (CIBERobn), Instituto Salud Carlos III, 28029 Madrid, Spain
[4]Department of Molecular Epidemiology, German Institute of Human Nutrition Potsdam-Rehbruecke, 14558 Nuthetal, Germany
[5]German Center for Diabetes Research, München-Neuherberg, 85764 Oberschleißheim, Germany
[6]Cardiovascular Research Translational Studies, Department of Clinical Sciences Malmö, Lund University, 20502 Malmö, Sweden
[7]Department of Cardiology, Skåne University Hospital, 21428 Malmö, Sweden
[8]Wallenberg Centre for Molecular Medicine, Lund University, 22362 Lund, Sweden
[9]Bioinformatics Unit, Department of Clinical Sciences Malmö, Lund University, 20502 Malmö, Sweden
[10]Latvian Biomedical Research and Study Centre, 1067 Riga, Latvia
[11]Institute of Nutritional Science, University of Potsdam, 14469 Potsdam, Germany
[12]Faculty of Biology, University of Latvia, 1004 Riga, Latvia
[13]Genetics and Diabetes Unit, Department of Clinical Sciences Malmö, Lund University, 20502 Malmö, Sweden
[14]Lead contact
*Correspondence: sgcalzon@unav.es (S.G.-C.), charlotte.ling@med.lu.se (C.L.)
https://doi.org/10.1016/j.xcrm.2025.102290

SUMMARY

Prediction of incident macrovascular events (iMEs) in individuals with type 2 diabetes (T2D) remains suboptimal. We aim to discover blood-based epigenetic biomarkers predicting iMEs in 752 newly diagnosed individuals with T2D, among whom 102 developed iMEs during follow-up. 461 DNA methylation sites, e.g., near *ARID3A*, *GATA5*, *HDAC4*, *IRS2*, and *TMEM51*, associate with iMEs. Using cross-validation, a methylation risk score (MRS) containing 87 sites predicts iMEs with an area under the curve (AUC) of 0.81 and an AUC of 0.84 for the combination of MRS and clinical risk factors, better than SCORE2-Diabetes (Systematic Coronary Risk Evaluation 2-Diabetes), UKPDS (United Kingdom Prospective Diabetes Study), Framingham, and polygenic risk scores (AUCs = 0.54–0.62). This epigenetic biomarker has a negative predictive value of 95.9% and improves the classification of iMEs with continuous net reclassification improvement (NRI) showing 90.2% improvement versus clinical factors. Atherosclerotic versus non-atherosclerotic aortas show 78 differentially methylated sites. We validate 32 sites in EPIC-Potsdam and 43 in OPTIMED cohorts, including an MRS (AUC = 0.80). Together, blood-based epigenetic biomarkers predict iMEs better than clinical risk factors, supporting its future clinical use.

INTRODUCTION

Type 2 diabetes (T2D) is a leading cause of death through cardiovascular disease (CVD).[1] Individuals with T2D have 2–4 times higher risk of CVD, including macrovascular events, such as myocardial infarction, angina, ischemic heart disease, or stroke, compared to the non-diabetic population after controlling for traditional risk factors namely age, obesity, smoking, dyslipidemia, and hypertension.[2,3] Identifying individuals with risk for macrovascular events is essential for disease prevention. However, prediction of macrovascular events in individuals with T2D is suboptimal. Risk scores for risk stratification of CVD exist,[4–9] but these showed moderate ability to stratify individuals with T2D into those who will develop CVD and those who will not.[10,11] For example, the UKPDS (United Kingdom Prospective Diabetes Study) risk calculator showed poor discrimination, overestimating CVD risk in diabetes,[11,12] although it is clinically used.[13] Consequently, there is an urgent need to discover new biomarkers, other than traditional risk factors, to improve the prediction of CVD and macrovascular events in individuals with T2D.

Genetic variants associated with CVD in individuals with T2D[14] showed limited improvement compared to clinical factors in prediction of CVD.[15] Additionally, epigenetics contribute to diabetic complications[16–19] and might explain the metabolic memory, where vascular stress persists after glucose normalization, thereby increasing complications.[20] Moreover, CVD associated with DNA methylation (DNAm) in

© 2025 The Author(s). Published by Elsevier Inc.
This is an open access article under the CC BY license (http://creativecommons.org/licenses/by/4.0/).

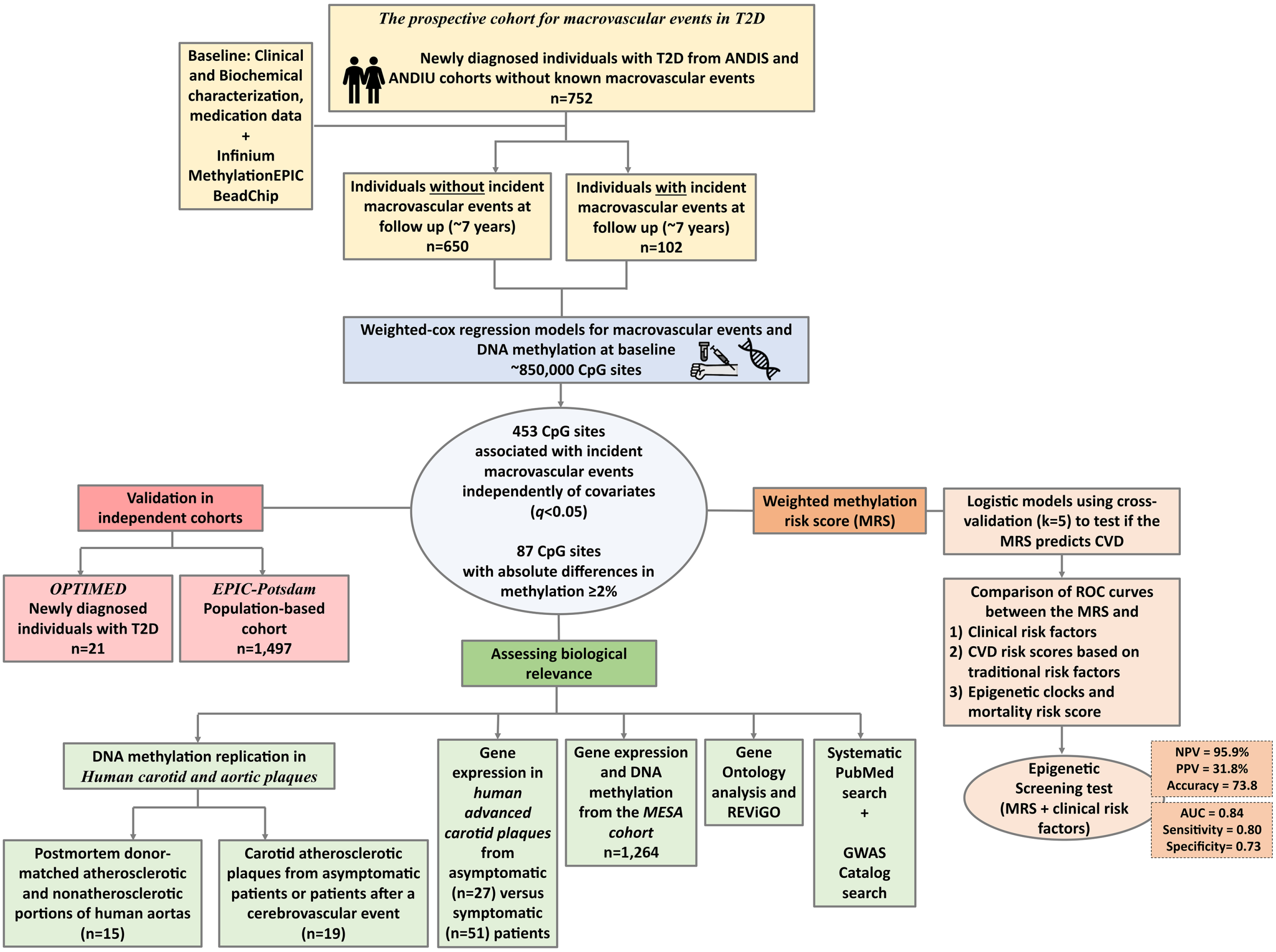

Figure 1. Flowchart of the overall study design

ANDIS, "All New Diabetics in Skane"; ANDIU, "All New Diabetics in Uppsala"; CVD, cardiovascular disease; T2D, type 2 diabetes; NPV, negative predicted value; PPV, positive predicted value.

case-control and prospective studies, mainly including non-diabetic individuals.[21,22] Notably, DNAm was associated with future diabetic retinopathy[16] and chronic kidney disease,[19] supporting the development of blood-based epigenetic biomarkers for the prediction of diabetic complications. However, prospective studies assessing whether DNAm predicts macrovascular events in newly diagnosed individuals with T2D are lacking. Such epigenetic biomarkers could provide useful clinical tools, identifying individuals with T2D at risk of developing CVD, making it possible to prevent, at an early stage, progression to macrovascular events.

Consequently, the main goal of this study was to identify blood-based epigenetic biomarkers of clinical relevance that predict macrovascular events including myocardial infarction, angina, stroke, or ischemic heart disease in newly diagnosed individuals with T2D using the prospective cohort for macrovascular events in T2D. These biomarkers could offer a valuable tool for precision medicine. We further investigated the potential biological relevance of the genes annotated to the identified blood-based epigenetic biomarkers by (1) performing a systematic literature search, (2) correlating DNAm with gene expression in human blood samples,[23] and (3) studying DNAm and gene expression in human plaques.[24–26] Finally, validation analyses were performed in two prospective cohorts, optimized program of personalized treatment of type 2 diabetes (OPTIMED) and the European Prospective Investigation into Cancer and Nutrition (EPIC)-Potsdam.

RESULTS

Epigenetic markers associate with future macrovascular events in T2D

Figure 1 shows the study design. Among 752 newly diagnosed individuals with T2D in the prospective cohort for macrovascular events in T2D free of macrovascular events at baseline, 102 developed macrovascular events, while 650 did not (censored/controls), during a mean and max follow-up of ~4 and 7 years, respectively. At baseline, individuals who developed macrovascular events were older, had lower glycated hemoglobin (HbA1c), higher systolic blood pressure, higher usage of

Table 1. Baseline clinical characteristics of the prospective cohort of macrovascular events in type 2 diabetes

	Prospective cohort for macrovascular events in type 2 diabetes (n = 752)					
	Controls (n = 650)		Incident macrovascular events (iMEs) (n = 102)			
	Mean (SD)	Min to Max	Mean (SD)	Min to Max	p value	AUC
Follow-up, years	3.9 (1.5)	0.01 to 7.2	–	–	–	–
Time to vascular event, years	–	–	2.0 (1.2)	0.01 to 5.5	–	–
Age	59.9 (11.5)	21 to 91	65.0 (10.1)	37 to 87	2×10^{-5}	0.63
Gender, males/females (%)	55/45	–	65/35	–	0.064	0.55
BMI	31.6 (5.3)	20.5 to 49.5	31.2 (6.2)	18.6 to 50.3	0.147	0.55
Hba1c, mmol/mol	64.1 (17.8)	30 to 129	59.4 (18.7)	35 to 128	0.0016	0.60
cg05575921, %methylation	78.5 (8.06)	49.6 to 90.24	74.28 (10.62)	50.12 to 87.80	0.0003	0.61
Drug therapy, %						
Glucose lowering	13	–	27	–	0.0002	0.57
Lipid lowering	20	–	25	–	0.261	0.52
Antihypertensives	40	–	57	–	0.0015	0.58
Cholesterol, mmol/L						
Total[a]	5.4 (1.3)	2.3 to 14.7	5.6 (1.1)	3.5 to 9.1	0.233	0.54
LDL[a]	3.4 (1.0)	0.8 to 8	3.6 (1.0)	1.3 to 6.8	0.412	0.47
HDL[a]	1.1 (0.3)	0.4 to 2.4	1.2 (0.4)	0.7 to 2.8	0.844	0.51
Triglycerides, mmol/L[a]	2.2 (1.5)	0.5 to 13.5	2.1 (1.4)	0.8 to 7.8	0.598	0.52
eGFR, mL/min/1.73 m^2[a]	91.9 (22.6)	44 to 182	89.1 (23.21)	40 to 167	0.301	0.53
Urinary albumin/creatinine ratio, mg/mmol[a]	2.2 (6.8)	0 to 82	7.0 (25.7)	0 to 182	0.0003	0.63
Systolic blood pressure, mm Hg	137 (16)	100 to 210	143 (16)	115 to 205	0.001	0.62
Diastolic blood pressure, mmHg	81 (10)	53 to 120	82 (10)	60 to 124	0.376	0.52

Phenotypes were measured in newly diagnosed individuals with diabetes from the ANDIS (All New Diabetics in Scania) and ANDiU (All New Diabetics in Uppsala) studies. p values were calculated with Mann-Whitney U test for continuous variables and Pearson's chi-square test for binary variables. $p < 0.05$ was considered significant. AUCs were calculated for each clinical variable using ROC curves and considering iMEs as outcome.
HbA1c, glycated hemoglobin; BMI, body mass index; HDL, high-density lipoprotein; LDL, low-density lipoprotein; eGFR, estimated glomerular filtration rate; SD, standard deviation; AUC, area under the curve.
[a]Missing values for total cholesterol (168 controls and 17 iMEs), LDL-cholesterol (186 controls and 21 iMEs), HDL-cholesterol (188 controls and 18 iMEs), triglycerides (212 controls and 26 iMEs), eGFR (149 controls and 19 iMEs), urinary albumin/creatinine ratio (194 controls and 33 iMEs), and systolic and diastolic blood pressure (83 controls and 21 iMEs).

diabetes medication and antihypertensives, and higher prevalence of smoking versus controls (Table 1).

To identify blood-based epigenetic biomarkers that associate with future macrovascular events in newly diagnosed individuals with T2D, we analyzed DNAm of 853,307 sites in blood taken at registration from the prospective cohort for macrovascular events in T2D. DNAm of 461 sites, annotated to 422 genes, associated with incident macrovascular events (iMEs) using a weighted-Cox regression model adjusted for age, gender, BMI, and HbA1c ($q < 0.05$, model 1) (Figure 2A; Table S1A). These sites are distributed across the genome (Figure S1A). We observed consistent results when adjusting for additional covariates, including cell composition, smoking, medications, and/or lipid profiles in models 2–10 (Figure 2A). In these models, methylation of 453 of 461 sites was associated with future macrovascular events based on $p = 7.10 \times 10^{-20}$ to 4.84×10^{-2} (Figure 2A; Table S1B). *AHRR* methylation (cg05575921), a reliable biomarker of smoking,[27] was among the 461 sites (Table S1A) but, understandably, did not remain significant when included as a covariate to adjust for smoking (Table S1B). After adjustment for baseline estimated glomerular filtrationr rate (eGFR) (n = 578) or urinary albumin/creatinine ratio (n = 521), and despite some missing data for these variables, methylation of 449 out of 461 sites was associated with future macrovascular events with $p = 6.9 \times 10^{-14}$ to 4.9×10^{-2} (Table S1B). When adjusting for diabetes medication, all 461 sites were associated with future macrovascular events with $p = 1.18 \times 10^{-10}$ to 2.2×10^{-2} (Table S1B).

MRS predicts future macrovascular events in T2D

We then tested whether blood-based DNAm, based on a combined methylation risk score (MRS), predicted future macrovascular events in newly diagnosed individuals with T2D. To select more robust methylation sites for inclusion in the MRS, we filtered the data in Table S1B to include sites with absolute methylation differences ≥2% between individuals with iMEs and controls. Subsequently, 87 methylation sites were included in the MRS (Figure 2B; Table S1C). Most of these 87 sites (~74%) were hypomethylated in individuals with iMEs versus controls, and their hazard ratio (HR) ranges from 0.4 to 2.1 per 1 SD increase in methylation (Table S1C). MethyltoSNP did not identify SNP-like patterns among these sites.[28]

The MRS including 87 sites was significantly different between individuals who developed macrovascular events and controls ($p < 2.2 \times 10^{-16}$) (Figure S1B). To assess if the MRS could

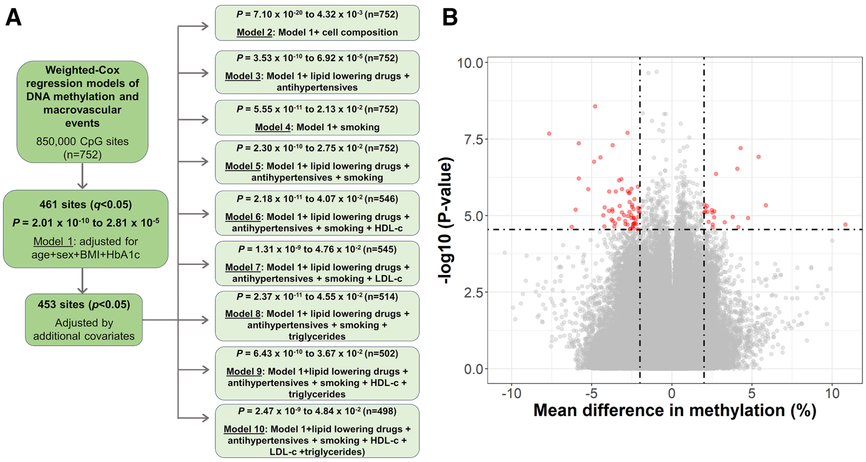

Figure 2. DNA methylation (DNAm) is associated with incident macrovascular events (iMEs) in newly diagnosed individuals with type 2 diabetes during ~7 years of follow-up

(A) Flow diagram of the 10 weighted-Cox regression models that were run using data from the 850K array to identify DNAm associated with iMEs. First, 461 sites were identified in model 1 after adjusting for age, gender, glycated hemoglobin (HbA1c), and body mass index (BMI) (FDR < 0.05). Then 453 out of these 461 sites were associated with iMEs based on $p = 7.10 \times 10^{-20}$ to 4.84×10^{-2}, after adjusting for additional covariates: cell composition, lipid-lowering drugs and antihypertensive drugs, smoking based on DNAm levels of *AHRR* (cg05575921), which is a biomarker of smoking, high-density lipoprotein-cholesterol levels (HDL-c), low-density lipoprotein-cholesterol levels (LDL-c), and triglyceride (TG) levels.

(B) Volcano plot showing the identified 87 sites in red associated with future macrovascular events (model 1, $q < 0.05$) and with absolute differences in DNAm ≥2% between individuals with iMEs and controls.

See also Figure S1; Table S1.

discriminate between individuals with iMEs and controls in the prospective cohort for macrovascular events in T2D, we ran 5-fold cross-validation using censoring and sampling weights to perform logistic models with MRS as an independent variable. As a comparison to the MRS, we performed a cross-validation analysis including only clinical risk factors for macrovascular events (age, gender, HbA1c, BMI, smoking, diabetes medication, lipid-lowering medication, and antihypertensives) as independent variables, to test how well these predict macrovascular events. We further combined the MRS and clinical risk factors, to test if this combination could improve prediction of macrovascular events. Predicted risks of each individual were obtained, and receiver operating characteristic (ROC) curves were generated with macrovascular event as outcome displaying area under the curves (AUCs) of 0.81 (95% confidence interval [CI]: 0.77–0.86) for the MRS, 0.69 (95% CI: 0.64–0.75) for only clinical risk factors, and 0.84 (95% CI: 0.79–0.88) for the combination of MRS and clinical risk factors (Figure 3A). When comparing these ROC curves, the AUC for the MRS (0.81) was significantly better than the AUC for only clinical risk factors (0.69, $p = 0.001$), but it was not significantly different than the combination of MRS and clinical risk factors (0.84, $p = 0.201$). The AUC for the combination of clinical risk factors and MRS (0.84) was also significantly better than the AUC including only clinical risk factors (0.69, $p = 1.7 \times 10^{-7}$) or the individual AUCs of these clinical risk factors (0.47–0.63) (Table 1). Since 13.6% of participants developed macrovascular events, while 86.4% did not, we made a precision-recall plot, commonly used for unbalanced data. Here, both precision (true positives/(true positives + false positives)) and recall or sensitivity (true positives/(true positives + false negatives)) were better for the MRS and the combination of MRS and clinical risk factors versus only clinical risk factors (Figure 3B).

We further compared the ability of the MRS, with some established CVD risk scores,[5–8] a polygenic risk score (PRS) including 204 SNPs associated with coronary artery disease (CAD) in T2D,[29,30] and epigenetic clocks of aging and mortality,[31–35] in predicting macrovascular events in the prospective cohort for macrovascular events in T2D (Figure 4). SCORE2-Diabetes[9] and UKPDS scores,[5] developed for predicting CVD in individuals with diabetes, generated AUCs of 0.62 and 0.54, respectively. Other risk scores for the general population, e.g., Framingham,[6] atherosclerotic cardiovascular disease (ASCVD),[7] and Multi-Ethnic Study of Atherosclerosis (MESA),[8] generated AUCs of 0.61–0.64, which were significantly worse in discriminating individuals who will develop macrovascular events from those who will not compared to the MRS or the combination of MRS and clinical risk factors (Figure 4A). The PRS generated an AUC of 0.61, significantly worse than the MRS (Figure 4A). AUCs of epigenetic clocks and mortality scores ranged from 0.61 to 0.68 for prediction of macrovascular events, which were significantly lower than the MRS or combination of MRS and clinical risk factors (Figure 4B). Clocks predictive of chronological and biological age (Bernabeu_cAge_2023,[31] ZhangQ_Age_2019,[34] Horvath_Age_2018,[32] Levine_PhenoAge_2018[33]) showed lower AUCs versus ZhangY_Mortality Risk_2017[35] aiming to estimate mortality risk. There is no overlap between methylation sites included in these epigenetic clocks and the ones in Table S1B.

Collectively, our results presented in Figures 3 and 4 support that the MRS is better than clinical risk factors in predicting future macrovascular events among individuals with T2D. However, since a combination of the MRS and clinical risk factors seems to have the best predictive capacity, we further explored the use of this combined biomarker tool. To be able to use the MRS together with clinical risk factors as an epigenetic screening test for prediction of macrovascular events, a cutoff point should be established to calculate predicting parameters. This cutoff point was calculated with the ROC curve generated with the MRS and clinical risk factors, using Youden index (point on ROC curve that has the largest vertical distance from the diagonal or chance line).[36] The optimal cutoff point was 0.023, with a sensitivity of 0.804 and specificity of 0.728, predicting good separation

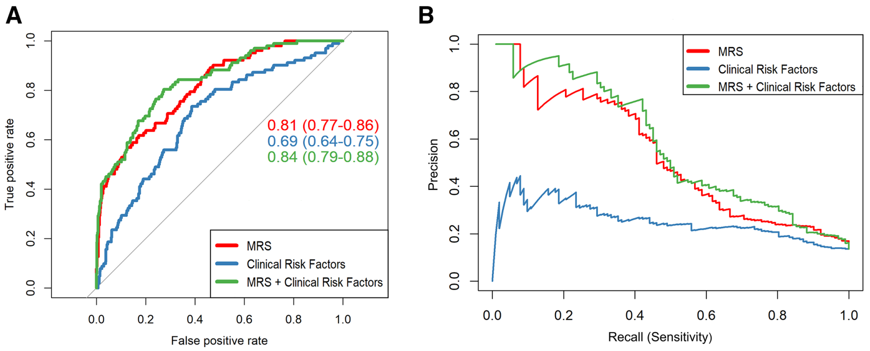

	MRS vs. Clinical Risk Factors	MRS vs. MRS + Clinical Risk Factors	MRS + Clinical Risk Factors vs. Clinical Risk Factors
P values AUCs	0.001	0.201	1.7×10^{-7}

Figure 3. A methylation risk score (MRS) predicts incident macrovascular events (iMEs) in newly diagnosed individuals with type 2 diabetes (T2D) during 7 years of follow-up

The MRS was generated using data from 87 methylation sites associated with iMEs and with absolute differences in DNA methylation ≥2% between individuals with iMEs and controls (see Figure 2B; Table S2B).

(A) ROC curves were generated with macrovascular events as the outcome and the predicted risks of each individual obtained using cross-validation (k = 5) for the MRS, for the clinical risk factors (age + gender + HbA1c + BMI + smoking [cg05575921 methylation] + diabetes medication + lipid-lowering medication + antihypertensives), and for the combination of both the MRS and clinical risk factors, separately. The MRS (AUC = 0.81, 95% CI: 0.77–0.86) could better predict iMEs in individuals with T2D compared to clinical risk factors (AUC = 0.69, 95% CI: 0.64–0.75, $p = 0.001$). The combined MRS and clinical risk factors (AUC = 0.84, 95% CI: 0.79–0.88) was even better at predicting iMEs compared to clinical risk factors (AUC = 0.69, $p = 1.7 \times 10^{-7}$), but it was not statistically better than the MRS alone (AUC = 0.81, $p = 0.201$).

(B) A precision-recall plot was also better for the MRS and the combination of MRS and clinical risk factors compared to only the clinical risk factors. This plot is commonly used for imbalanced data, in our case 13.6% iMEs and 86.4% controls, where the precision can be defined as true positives/(true positives + false positives) and recall or sensitivity is calculated as true positives/(true positives + false negatives).

between individuals with T2D who developed macrovascular events and those who did not (Figure 5A). Consequently, this epigenetic test can correctly identify 80.4% of individuals who will develop macrovascular events, leading to only 19.6% individuals classified as false negatives (Figure 5B). When calculating metrics of reclassification, the epigenetic test demonstrated significant improvements over the standard model including clinical risk factors. Using the cutoff = 0.023 to define risk categories, the categorical net reclassification improvement (NRI) indicated 28.2% improvement in correctly classifying individuals into both categories (NRI = 0.282, 95% CI: 0.18–0.39, $p < 0.001$), whereas the continuous NRI revealed a substantial 90.2% enhancement in the model's ability to accurately predict risk on a continuous scale (NRI = 0.902, 95% CI: 0.71–1.09, $p < 0.001$). The integrated discrimination improvement (IDI) showed a 17.9% increase in the model's capacity to discriminate between those who will and will not experience macrovascular events (IDI = 0.179, 95% CI: 0.13–0.23, $p < 0.001$).

Considering prevalence, which was 13.6% (100*(102/752)), this epigenetic screening test demonstrated a very accurate detection of individuals free of future macrovascular events among newly diagnosed individuals with T2D (negative predictive value [NPV] = 95.9%) (Figure 5C). Therefore, when used for screening for macrovascular events of asymptomatic individuals, a negative result gives important information, such as reduced need for follow-up of these patients with T2D as intensively, saving time and costs for healthcare systems. However, this test had moderate false-positive rate and did not fully reliably identify iMEs in asymptomatic people with T2D (positive predictive value [PPV] = 31.8%) (Figure 5C). Thus, while the NPV is excellent, the PPV should be improved by further analyses. Together, these data, summarized in Figure 5D, support potential clinical use of blood-based epigenetic biomarkers for the prediction of macrovascular events in T2D.

Taking the cutoff point = 0.023 into account, we split the data into two groups: those with low values of the epigenetic test ($n = 496$) and those with high values ($n = 256$). The Kaplan-Meier curve, for survival analysis for future development of macrovascular events in individuals with T2D, showed a significantly higher survival proportion for individuals with low values of MRS and clinical risk factors versus those with high values ($p = 8.65 \times 10^{-24}$, Figure 5E). A weighted-Cox analysis revealed that the 256 individuals with high values of MRS and clinical risk factors have a greater risk of developing macrovascular events with HR = 9.33 (95% CI: 5.53–15.76, $p = 6.42 \times 10^{-17}$) versus those with low values during ∼7 years follow-up.

Biological relevance of DNAm associated with iMEs in T2D

To better understand the biological relevance of the 453 methylation sites associated with iMEs in T2D, we performed gene ontology (GO) and REVIGO analyses.[37] We identified 11 biological processes ($p < 0.01$), including regulation of protein oxidation, embryonic heart tube development, and transport along microtubule (Table S2A).

We further performed a systematic literature search of 64 genes annotated to the 87 sites in the MRS, using each gene symbol and the following terms: myocardial infarction, angina, ischemic heart disease, stroke, or CVD (Table S2B). Here, 39 of 64 genes (61%) were associated with any of these terms (Figure 6A). Specifically, 17 genes (27%) have been associated with myocardial infarction (e.g., *HDAC4*, *HIPK3*, and *MEIS1*), 16 genes (25%) with ischemic heart disease (e.g., *GATA5*, *IRS2*, and *P2RY13*), 15 genes (23%) with stroke (e.g., *LEF1*, *SOX2*, and *SPARCL1*), one with angina (i.e., *HDAC4*), and 38 genes (59%) with the terms vascular or CVD (e.g., *MCF2L* and *CXXC5*). We used GWAS Catalog to determine whether SNPs annotated to these 64 genes have been associated with CVD or related traits, and we found SNPs annotated to *MAP7D3*,

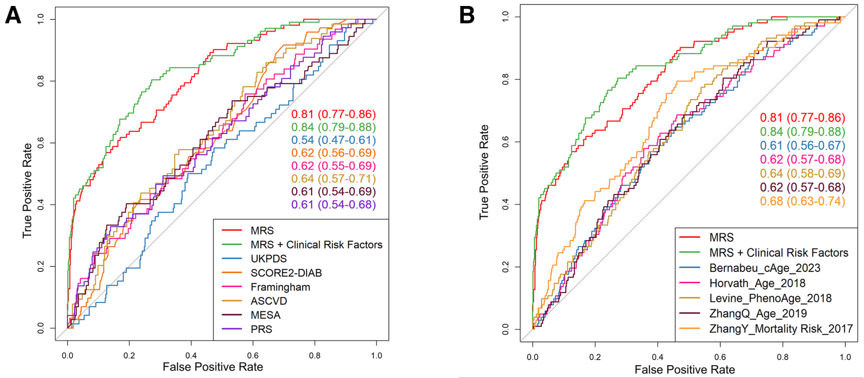

P values AUCs	UKPDS	SCORE2-DIAB	Framingham	ASCVD	MESA	PRS
MRS	6.3×10^{-11}	2.3×10^{-6}	1.3×10^{-5}	5.0×10^{-5}	6.8×10^{-6}	3.5×10^{-6}
MRS + Clinical Risk Factors	1.1×10^{-12}	9.6×10^{-8}	9.3×10^{-7}	3.8×10^{-6}	4.6×10^{-7}	2.0×10^{-7}

P values AUCs	Bernabeu_ cAge_2023	Horvarth_ Age_2018	Levine_Pheno Age_2018	ZhangQ_Age _2019	ZhangY_ MortalityRisk _2017
MRS	5.5×10^{-8}	1.1×10^{-7}	1.1×10^{-7}	6.3×10^{-8}	2.9×10^{-5}
MRS + Clinical Risk Factors	1.2×10^{-12}	3.7×10^{-12}	5.3×10^{-12}	1.1×10^{-12}	2.0×10^{-7}

Figure 4. Comparison between the methylation risk score (MRS) and established cardiovascular (CVD) risk scores and epigenetic clocks of aging and mortality at predicting incident macrovascular events (iMEs) in individuals with type 2 diabetes (T2D)

(A) The MRS (AUC = 0.81) and the combination of the MRS and clinical risk factors (age + gender + HbA1c + BMI + smoking [cg05575921 methylation] + diabetes medication + lipid-lowering medication + antihypertensives) (AUC = 0.84) were better at predicting iMEs compared to previously established CVD risk scores, including the SCORE2-Diabetes calculator,[9] the UKPDS score,[5] Framingham,[6] ASCVD,[7] and MESA[8] (AUCs = 0.54–0.64), and compared to a polygenic risk score (PRS) of coronary artery disease (CAD) developed in T2D population (AUC = 0.61).

(B) The MRS (AUC = 0.81) and the combination of the MRS and clinical risk factors (AUC = 0.84) were better at predicting iMEs compared to previously established epigenetic clocks of aging and mortality, including ZhangQ_Age_2019,[34] Horvath_Age_2018,[32] Bernabeu_cAge_2023,[31] Levine_PhenoAge_2018,[33] and ZhangY_Mortality Risk_2017[35] (AUCs = 0.61–0.68).

See also Table S7.

PPFIA1, *NYAP2*, and *KSR2* associated with CAD and *RUFY4* associated with ischemic stroke (Table S2C; Figure 6B). Overall, 46 of 64 genes (72%) have been associated with CVD and 18 (28%) have not; however, 6 of these 18 genes were associated with diabetes (Figure S1C).

Next, because DNAm may regulate gene expression,[38–40] we tested whether DNAm correlated with gene expression using Infinium 450K array data from the MESA cohort ($CD14^+$ monocytes from blood of 1,264 individuals, GEO: GSE56047).[23] Of the 87 sites in our MRS, 61 were available on the 450k array. Among these, 39 were annotated to 38 genes, and 22 lacked annotation. We found that the expression of 14 genes (e.g., *HDAC4*, *MEIS1*, *GATA5*, *CXXC5*, *TMEM51*, and *ARID3A*) correlated with DNAm in monocytes (Table S3; Figure 6C).

We then assessed whether blood-based DNAm of the 453 sites associated with iMEs also plays a role in target tissues underlying the disease, including human aortic and carotid plaques, using publicly available 450k array data covering 305 of 453 sites (STAR Methods). Among these 305 sites, 102 ($p < 0.05$) and 78 ($q < 0.05$) sites showed differential DNAm between atherosclerotic and non-atherosclerotic portions of aortas (Table S4A). Thirty-four of 78 sites are among the 87 sites in the MRS (Figure 6D). We found 8 sites differentially methylated between symptomatic and asymptomatic carotid plaques at $p < 0.05$, but none had false discovery rate (FDR) < 5% (Table S4B). cg16245698 was differentially methylated in both aortic and carotid samples and is among the sites in the MRS.

Finally, we explored whether the expression of the 64 genes annotated to the 87 sites in the MRS, and associated with iMEs, shows differential expression in human advanced carotid plaques from asymptomatic ($n = 27$) versus symptomatic ($n = 51$) patients. Interestingly, 4 genes, including *TMEM51* and *ARID3A*, showed differential expression in symptomatic versus asymptomatic carotid plaques (Figure 6E), further supporting a biological relevance of the genes annotated to the methylation sites associated with iMEs in target tissues for the disease.

Validation of methylation markers associated with iMEs in T2D

Finally, we performed validation analyses of the methylation markers associated with iMEs in the prospective cohort for macrovascular events in T2D (Table S1B), using MethylationEPIC BeadChip data from blood of two prospective cohorts, OPTIMED[41] and EPIC-Potsdam.[42] We included 21 newly diagnosed individuals with T2D from OPTIMED, of whom 10 developed macrovascular events within ∼11 years follow-up (Figure S2A; Table S5A). The EPIC-Potsdam cohort includes 1,497 individuals from the general population, of whom 427 developed macrovascular events within ∼8 years (Table S5B). Using weighted-Cox regression models adjusted for age, gender, BMI, and HbA1c, 43 methylation sites were validated in OPTIMED (Table S6A) and 32 in EPIC-Potsdam (Table S6B).

Validation of MRS predicting iMEs in T2D

Among the 87 sites in the MRS (Table S1C), five were associated with iMEs in OPTIMED (Table S6C). We generated an MRS, including these five sites (MRS_{5sites}), which significantly differed between individuals with iMEs and controls, in both OPTIMED ($p = 3.8 \times 10^{-4}$, Figure S2B) and the prospective cohort for macrovascular events in T2D ($p = 1.05 \times 10^{-15}$, Figure S2C). Individuals who developed macrovascular events showed 2.5- to 3.5-fold increased risk versus controls per 1 SD increase in MRS_{5sites}, with HR = 3.47 ($p = 0.016$) in OPTIMED and HR = 2.51 ($p = 5.29 \times 10^{-15}$) in the prospective cohort for macrovascular events in T2D (Figures S2B and S2C). Next, we ran cross-validation using logistic models with the MRS_{5sites} combined with clinical risk factors and generated ROC curves with macrovascular events as the outcome. These displayed AUC = 0.80 (95% CI: 0.60–0.99) for OPTIMED (Figure S2D)

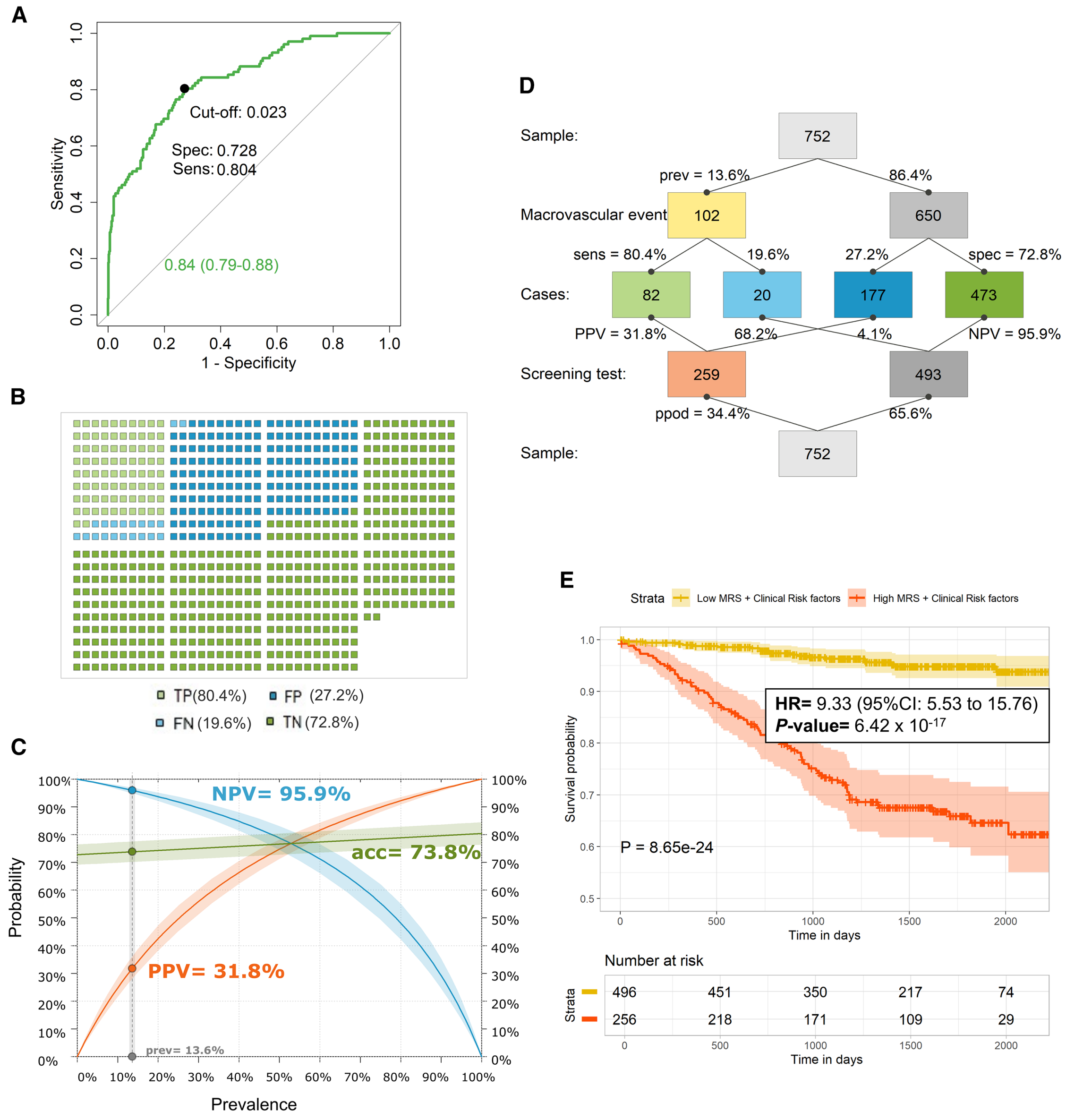

Figure 5. Prediction of macrovascular events in individuals with type 2 diabetes (T2D) using an epigenetic screening test, including the methylation risk score (MRS) and clinical risk factors

(A) The optimal cutoff point, which was 0.023, was calculated using the ROC curve for the combined MRS and clinical risk factors (AUC = 0.84) with the Youden index (point on ROC curve that has the largest vertical distance from the diagonal or chance line) giving a sensitivity (Sens) of 0.804 and a specificity (Spec) of 0.728.

(B) Icon plot showing 80.4% (82/102) as true positives (TP), 19.6% (20/102) as false negatives (FN), 72.8% (473/650) as true negatives (TN), and 27.2% (177/650) as false positives (FP).

(C) Curve plot using prevalence of macrovascular events (13.6%) and showing accuracy (acc) (73.8%), negative predictive value (NPV) (95.9%), and positive predictive value (PPV) (31.8%).

(D) Summary diagram of the epigenetic screening test for the combined MRS and clinical risk factors showing all the indicated predicting parameters.

(E) Kaplan-Meier macrovascular event-free survival analysis. Data were split into two groups based on the cutoff point (0.023): those with low predicted values of MRS and clinical risk factors (n = 496) and those with high values (n = 256). The Kaplan-Meier curve, for survival analysis for future development of macrovascular events in individuals with T2D, displays that the survival proportion was significantly higher for individuals with low values of MRS and clinical risk factors (age + gender + HbA1c + BMI + smoking [cg05575921 methylation] + diabetes medication + lipid-lowering medication + antihypertensives) compared to those individuals with high values (log-ranked $p = 8.65 \times 10^{-24}$). Hazard ratio (HR) with 95% confidence interval (CI), based on a weighted-Cox analysis, is also displayed showing that the 256 individuals with high values of MRS and clinical risk factors have a greater risk of developing macrovascular events with an HR of 9.33 (95% CI: 5.53–15.76, $p = 6.42 \times 10^{-17}$) compared to 496 individuals with low values during ~7 years of follow-up.

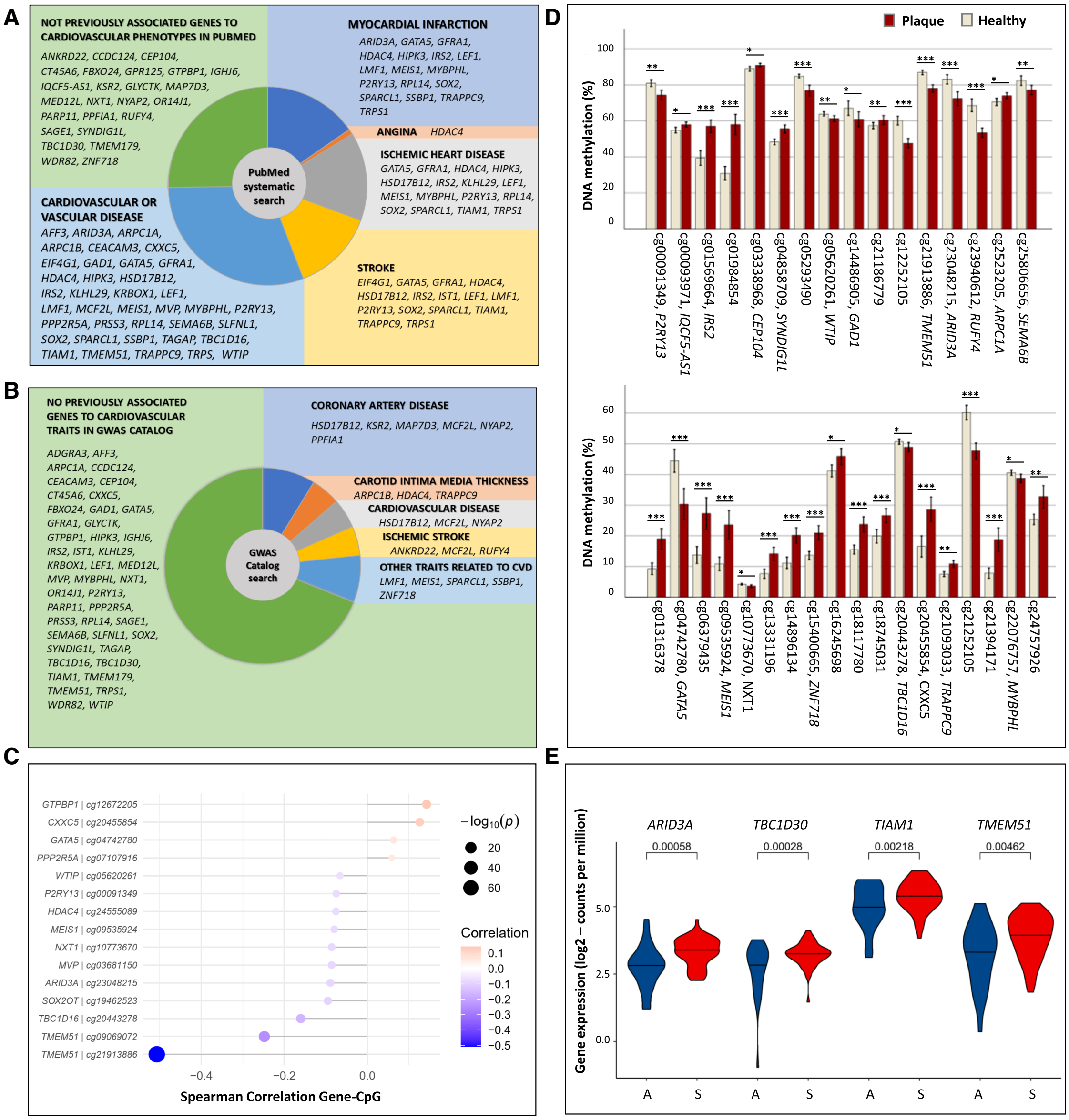

Figure 6. Biological relevance assessment of methylation sites associated with incident macrovascular events (iMEs) in individuals with type 2 diabetes

(A) Phenotype wheel of the genes annotated to the 87 methylation sites in the methylation risk score (MRS) based on a systematic PubMed search: the figure shows the 64 genes annotated to the 87 methylation sites associated with iMEs in our cohort and included in the MRS, and their associated phenotypes according to the following PubMed search: "gene AND Vascular Disease OR Cardiovascular Disease," "gene AND Myocardial Infarction," "gene AND Stroke," "gene AND Ischemic Heart Disease," "gene AND Angina." 39 of the 64 genes (61%) were previously associated with at least one of the phenotypes of cardiovascular (CVD) considered, and 25 (39%) were not previously associated with these phenotypes.

(B) GWAS Catalog traits wheel of the genes annotated to the methylation sites included in the MRS: the figure shows the 64 genes annotated to the 87 methylation sites associated with iMEs in our cohort and included in the MRS, and CVD-related traits to which SNPs, annotated to the same 64 genes, were associated based on GWAS Catalog (accessed Nov. 2023). Main traits considered: CVD, coronary artery disease, carotid intima media thickness, and ischemic stroke. Other CVD traits were considered: left ventricular mass to end-diastolic volume ratio, idiopathic dilated cardiomyopathy, vascular endothelial function, left ventricular mass index, and PR interval.

(C) Correlations between DNA methylation (DNAm) and gene expression in monocytes from blood of the Multi-Ethnic Study of Atherosclerosis (MESA) cohort. The figure shows 14 correlations ($p < 0.05$) in monocytes from $n = 1{,}264$ individuals among the 39 sites out of 87 included in the MRS that are available on the 450k array and are annotated to 38 unique genes. Spearman's correlation coefficients between genes and methylation sites are displayed on the x axis.

(legend continued on next page)

and 0.78 (95% CI: 0.73–0.83) for the prospective cohort for macrovascular events in T2D (Figure S2E).

DISCUSSION

In newly diagnosed individuals with T2D, we identified and cross-validated a blood-based epigenetic biomarker that predicts the risk for the first macrovascular event, independently of and in combination with clinical risk factors. This epigenetic screening test seems to be one of the most reliable prognostic tools for discrimination of the risk of macrovascular events in individuals with T2D, which could facilitate personalized follow-up and treatment from the onset of diabetes. Precision medicine, preventing macrovascular events in high-risk T2D populations, would reduce patient suffering and the economic burden of CVD.[43]

Individuals with T2D show heterogeneity in cardiovascular risk, and it remains unclear why some develop macrovascular events whereas others don't.[43] However, hyperglycemia and insulin resistance accelerate atherosclerosis, increasing the risk of macrovascular events. Additional risk factors for macrovascular events include hypertension, obesity, smoking, and dyslipidemia. Lifestyle changes, promoting physical activity, healthy diets, weight reduction, quitting smoking, reducing alcohol consumption, intensive glycemic control, and cardioprotective therapies, are tools used for CVD prevention and treatment.[43–45]

European guidelines classify individuals with diabetes into high or very high CVD risk based on the presence of target organ damage, presence of $\geq$3 risk factors or a T2D duration $\geq$10 years.[44,45] However, this is not accurate and useful for newly diagnosed individuals with T2D since, as can be seen in our study, most of them do not seem to have organ damage at diabetes diagnosis, for example, only one had retinopathy (data not shown) and/or have few risk factors due to the early stage of disease. Additionally, existing risk scores for the prediction of CVD showed moderate ability in stratifying individuals with T2D into those who will develop CVD and those who will not.[5,10,11] In our study, SCORE2-Diabetes and UKPDS scores and a PRS showed poor prediction of macrovascular events in individuals with T2D. Some studies identified biomarkers, other than traditional risk factors, showing moderate improvement in predicting CVD in diabetes. For example, N-terminal pro-B-type natriuretic peptide (NT-proBNP) and high-sensitivity cardiac troponin T (hs-TnT) showed AUCs of 0.72–0.79 but showed limited improvement in reclassification.[46,47] Circulating $CD34^+$ stem cells and amino acids predicted adverse vascular outcomes in T2D moderately.[48,49] Nevertheless, their modest improvements in risk discrimination are not sufficient to change decision-making in clinical practice. In contrast, our epigenetic screening test showed better risk prediction of macrovascular events in individuals with T2D, in terms of discrimination (AUC = 0.84) and NPV (95.9%), meeting requirements for biomarkers intended to enable individualized risk stratification in clinical practice. Although we validated an MRS including five sites in OPTIMED, further validation and optimization are needed.

Stratifying individuals with T2D at high and low risk for developing macrovascular events at an early stage would allow implementation of tailored preventive and therapeutic strategies to prevent and/or delay disease progression, thus reducing mortality and health-related costs. Diabetes was responsible for 6.7 million deaths in 2021 and caused $\geq$966 billion US dollars in health expenditure.[50] Based on theoretical evidence,[51,52] using our epigenetic screening test, estimated to cost ~$200/sample, may decrease the costs and deaths related to macrovascular events. However, real-world implementation and long-term studies are needed to quantify savings and mortality reductions. Individuals with T2D whose MRS in combination with clinical risk factors was >0.023 were likely to develop macrovascular events over ~7 years. They could be provided more personalized care, with healthcare visits promoting optimal medical therapy, improved glycemic control, and lifestyle changes.[53] For instance, in individuals with T2D and higher cardiovascular risk, SGLT2 (sodium-glucose co-transporter 2) inhibitors showing cardiovascular benefits,[54] along with intensive lipid-lowering and antihypertensive medications, could be recommended to reduce CVD.[44,54] The high NPV of our epigenetic screening test could identify individuals with T2D at lower risk for macrovascular events, allowing personalized treatment, optimized healthcare costs, and reduction of therapy-related side effects and patients' worries. The relatively moderate PPV observed is likely explained by the relatively low prevalence of macrovascular events in our cohort (13.6%), the early disease stage of participants newly diagnosed with T2D, and the follow-up period, which may be insufficient for a substantial number of macrovascular events to develop from diagnosis. As expected, under Bayes' theorem, a lower event prevalence reduces the PPV even when a model's sensitivity and specificity are strong. Subsequently, to evaluate the discrimination performance of the model independently of prevalence, we included widely accepted metrics, e.g., sensitivity, specificity, NRI, and IDI, all showing good performances.

The 87 sites associated with iMEs and included in the MRS are annotated to genes plausibly involved in CVD. For instance, *IRS2* was upregulated in males with T2D and CVD[55] and Irs2-silencing aggravated atheroma development.[56] Moreover, HDAC4, an epigenetic enzyme, associated to endothelial cell functions and angiogenesis,[57] was linked to ischemic stroke and

(D) The 34 sites differentially methylated between aortic plaques and healthy aortic tissues. The figure shows the 34 sites differentially methylated between aortic plaques (n = 15) and healthy aortic tissue (n = 15) after correction for multiple testing (FDR < 5%), which are among the 87 sites included in the MRS (59 out of 87 were covered by the 450k array). A paired t test comparison of DNAm among aortic healthy versus plaque tissue samples in the 15 subjects was applied. Plaques are shown in red and healthy tissues in beige. Error bars ±2 SE. *q < 0.05, **q < 0.01, and ***q < 0.001 for aortic plaques versus healthy aortic tissues.
(E) Genes differentially expressed in human advanced carotid plaques from asymptomatic (n = 27) versus symptomatic (n = 51) patients. The figure shows the significant differences in expression (FDR < 0.05) among the 64 genes annotated to the 87 methylation sites associated with iMEs. Raw p values are presented in the figure, and the FDR adjusted values are: q = 0.021 for *ARID3A*, q = 0.019 for *TBC1D30*, q = 0.035 for *TIAM1,* and q = 0.049 for *TMEM51*.
See also Figure S3; Tables S2, S3, and S4.

post-stroke recovery.[58] *HIPK3* encodes a circular RNA implicated in CVD,[59] being upregulated in individuals with T2D with retinal endothelial dysfunction,[60] and its knockdown attenuated myocardial fibrosis and enhanced cardiac function.[61] *GATA5* was associated with endothelial dysfunction and hypertension.[62] LEF1, a transcription factor, impacts the cardiac pathological hypertrophic remodeling process.[63] Additionally, several identified blood-based epigenetic biomarkers had differential methylation in atherosclerotic plaques, a relevant tissue for macrovascular events. These include sites in genes linked to CVD e.g., *CXXC5*, *GAD1*, *GATA5*, *IRS2*, and *SOX2*.[55,56,62] We also found that DNAm at some sites (e.g., *HDAC4*, *GATA5*, *CXXC5*, *TMEM51*, and *ARID3A*) correlated with gene expression in monocytes from the MESA cohort.[23] Interestingly, we found that *TMEM51* and *ARID3A* were also concordantly differentially expressed in symptomatic human advanced carotid plaques (Figure S3). These findings support that genes annotated to the epigenetic biomarkers we identified in individuals with T2D have a biological role linked to CVD. Although one of the main mechanisms of the epigenome is to regulate cell-specific gene expression, and the DNAm pattern is largely cell type specific, a few studies also found that DNAm of some sites in blood mirror the methylation pattern in target tissues, including atherosclerotic plaques.[39,64–68] As atherosclerotic plaques are composed of smooth muscle cells infiltrated with media from the vascular wall and inflammatory cells from blood, these infiltrated cells could influence the methylation pattern in atherosclerotic plaques. This could imply a higher expected concordance, compared to other tissues.

This prospective study addresses a previously underexplored association between DNAm and iMEs in newly diagnosed individuals with T2D, using an epigenome-wide association study (EWAS). A different study analyzed DNAm (450k) in relation to incident coronary heart disease (iCHD) in the general population.[22] The methylation sites associated with iCHD in this general population[22] were different from the sites associated with iMEs in T2D in our study. However, both our study and the study by Agha et al. found DNAm in *TRAPPC9*, *PTPRN2*, and *STAMBPL1* associated with iMEs in T2D, and iCHD in the general population. Additionally, *TRAPPC9* DNAm is included in our MRS and was different between aortic plaques and healthy aortic tissues. TRAPPC9 impacts nuclear factor κB signaling, and a SNP annotated to *TRAPPC9* was associated with intracerebral hemorrhage.[69] Other studies explored the association between DNAm and CVD risk in type 1 diabetes without finding any significant results after correction for multiple testing,[70] or in a general population cohort using methylation-derived protein EpiScores.[71] However, these studies employed different designs, different populations, or only analyzed specific methylation sites. Additionally, DNAm of *PTPRN2*, *LMF1*, and *KDM6A* differed in blood from 10 individuals with T2D with cardiovascular events at baseline versus 10 without cardiovascular events at baseline.[72] Interestingly, methylation in these genes was also associated with iMEs in our population. However, their discovery cohort differed from ours, considering prevalent cardiovascular events at baseline in a small sample size, rather than focusing on iMEs in newly diagnosed patients with T2D. Compared to previous studies, our work contributes in several ways: i.e., discovery of blood-based DNAm associated with future macrovascular events in newly diagnosed individuals with T2D, an epigenetic biomarker tool predicting iMEs better than known clinical risk scores, e.g., SCORE2-Diabetes and UKPDS, validation of numerous sites in independent prospective cohorts, and data supporting a biological relevance of identified methylation markers in human plaques.

This study has some strengths. Analyzing blood-derived epigenetic biomarkers is cost-effective, easy, minimally invasive, and safe, making it suitable for large-scale population screening and risk stratification. Correlations between expression and DNAm in blood, and analyses of DNAm and expression in plaques, strengthen the biological plausibility of the identified blood-based biomarkers. Our study used k-fold cross-validation, a valid approach to evaluate prediction accuracy in a single dataset by fitting the model to multiple parts of the data, reducing the risk of overfitting,[73] since it provides a relatively robust measure of a model's predictive performance by allowing the evaluation on multiple train-test splits. The reliability of our findings was further strengthened by validation in independent cohorts. The method used for the analysis of DNAm has strengths. By biological and technical validation, we previously showed that the method is stable and reliable.[39,74–76] Additionally, the EPIC array offers an efficient platform, optimized for high-throughput analysis and integration with existing EWAS databases.

In conclusion, our study discovered a blood-based epigenetic screening test that clearly can discriminate between newly diagnosed individuals with T2D who will develop macrovascular events and those who will not. The predictive capacity of this epigenetic biomarker was much better than established clinical risk scores, supporting its future use for precision medicine in T2D.

Limitations of the study

Lack of validation in different ethnicities is a limitation. Additionally, future studies should provide external validation of the full predictive model in other large prospective cohorts with DNAm analyzed in blood at T2D diagnosis (data currently not available).

Integrating epigenetic biomarkers into clinical practice poses some challenges. Studies by our group and others showed that environmental factors, e.g., diet, physical activity, medication, and aging, can modify DNAm of specific genomic regions.[38,68,77–80] Subsequently, future longitudinal studies are needed to confirm the temporal stability and predictive robustness of the identified epigenetic biomarkers. However, as DNAm is inherited through cell divisions, it offers a biomarker that can persist over time.[38] Furthermore, existing approved epigenetic cancer tests, e.g., analyzing *SEPT9* methylation,[81] support the use of epigenetic biomarkers in clinical practice.

Further limitations include lack of physical activity, dietary patterns, alcohol consumption, and fatal cardiovascular events in our cohort. All analyses were adjusted for gender; however, gender-specific effects were not explored further due to the moderate sample size and limited number of incident cases (n = 102), which made stratified analyses less feasible.

Although the EPIC array platform is robust, some genomic regions have limited coverage, and one may hence consider sequencing to cover full methylomes.[82] But due to higher cost and need for large server capacity, this method is less

feasible for larger cohorts. Furthermore, high dimensionality of sequencing data increases the multiple testing burden, necessitating much larger sample size to achieve sufficient statistical power after correction for multiple testing.[78]

Finally, although the identified blood-based epigenetic biomarkers showed biological relevance in plaques, this tissue contains several cell types,[83] and differences in cell composition may impact on our results. Hence, future epigenetic studies in single cells from plaques should be prioritized. Functional annotation and further mechanistic studies are also needed to determine whether the blood-based epigenetic biomarkers identified in this study play a causal role in disease development.

RESOURCE AVAILABILITY

Lead contact

Further information and requests for resources and reagents should be directed to and will be fulfilled by the lead contact, Charlotte Ling (charlotte.ling@med.lu.se).

Materials availability

This study did not generate new unique reagents.

Data and code availability

- The DNAm data from ANDIS and ANDiU and OPTIMED have been deposited in the LUDC repository (https://www.ludc.lu.se/resources/ludc-repository): accession number LUDC2023.12.1. Data are available upon request through the repository portal and acceptance by a review board. The DNAm data cannot be publicly deposited due to ethical and legal restrictions related to the Swedish Biobanks in Medical Care Act, the Personal Data Act, and the European Union's General Data Protection Regulation (GDPR) and Data Protection Act. DNAm data from atherosclerotic/nonatherosclerotic portions of aortas (GEO: GSE46401) and from asymptomatic/symptomatic carotid plaques (GEO: GSE66500) are deposited and available in Gene Expression Omnibus (https://www.ncbi.nlm.nih.gov/geo/). The human data from the CPIP biobank are protected due to privacy laws and would be shared in group level upon request from a qualified academic investigator for the sole purpose of replicating the procedures and results presented in the article and provided that the data transfer is in agreement with European Union legislation on the general data protection regulation and decisions by the ethical review board of Sweden, Region Skåne, and the Lund University. Professor Isabel Goncalves (isabel.goncalves@med.lu.se) may be contacted for data access from the CPIP biobank. Data regarding living subjects cannot be publicly available due to the sensitive nature of the data regulated by GDPR. DNAm and gene expression from the MESA cohort are publicly available (GEO: GSE56047). Information on data access and contact details for EPIC-Potsdam can be obtained at https://www.dife.de/en/research/cooperations/epic-study/.
- This article doesn't report original code.
- Any additional information required to reanalyze the data reported in this article is available from lead contact upon request.

ACKNOWLEDGMENTS

We thank the participants in ANDIS, ANDiU, CPIP, OPTIMED, and EPIC-Potsdam and the study personnel involved in data collection, processing, and management, as well as Professor Leif Groop, Maria Sterner, Gabriella Gremsperger, and Dr. Mats Martinell for valuable support, and SCIBLU genomics facility at Lund University for technical support with DNAm analysis. This study was supported by grants from the Swedish Research Council (2018-02567 and 2021-00628 to C.L., 2015-02523, 2019-01260, and 2023-02368 to I.G., 2024-02761 to A.E., and 2020-02191 to E.A./ANDIS), Swedish governmental funding of clinical research/Region Skåne (ALF, C.L., I.G., A.E., and ANDIS), Skåne University Hospital Funds, Strategic Research Area Exodiab (Dnr 2009-1039), Novo Nordisk Foundation (C.L. NNF19OC0057415 and E.A. NNF21OC0070457), Swedish Foundation for Strategic Research (Dnr IRC15-0067), Swedish Society for Medical Research (CG-22-0254 to A.E.), LeDucq Foundation Network of Excellence: CHECKPOINT ATHERO (22CVD02 to I.G.), Knut and Alice Wallenberg Foundation, Medical Faculty at Lund University and Region Skåne (N/A to A.E.), Swedish Diabetes Foundation (C.L.), Swedish Heart and Lung Foundation (C.L. nos. 20160602 and 20241100, E.A. no. 20220606, I.G. nos. 20200403 and 20230257, and A.E. nos. 20220044 and 20220284), and H2020-Marie-Curie grant (no. 706081, EpiHope), and ANDIS was also funded by the Faculty of Medicine (Lund University) and Vinnova Swelife. S.G.-C. was supported by a postdoctoral fellowship (Juan de la Cierva-Incorporación, IJC2019-040796-I). Work in EPIC-Potsdam was supported by a grant from the German Federal Ministry of Education and Research and the State of Brandenburg (DZD; 82DZD00302 and 82DZD03D03).

AUTHOR CONTRIBUTIONS

C.L. initiated the project. C.L., S.G.-C., E.A., and I.G. designed experiments and/or analyses and interpreted data. M.M., M.B., J.K., M.B.S., A.E., and E.A. initiated clinical studies and collection of data. S.G.-C., A.P., A.M., F.E., J.S., and M.M. performed statistical and bioinformatic analyses. C.L., S.G.-C., and A.M. drafted the manuscript. All authors read and edited themanuscript.

DECLARATION OF INTERESTS

A.E. received consulting fees from Novo Nordisk, Sanofi, Amarin, and Amgen with no relationship to the current study.

STAR★METHODS

Detailed methods are provided in the online version of this paper and include the following:

- KEY RESOURCES TABLE
- EXPERIMENTAL MODEL AND STUDY PARTICIPANT DETAILS
  - Discovery cohort: The prospective cohort for macrovascular events in T2D
  - Validation cohorts
  - Carotid plaque imaging project (CPIP) biobank
  - The Multi-Ethnic Study of Atherosclerosis (MESA) cohort
- METHOD DETAILS
  - Phenotypes measurements in the prospective cohort for macrovascular events in T2D
  - DNAm profiling in the prospective cohort for macrovascular events in T2D
  - Gene ontology of methylated sites associated with incident macrovascular events
  - DNAm in human plaques
  - RNA-sequencing of human carotid plaques
- QUANTIFICATION AND STATISTICAL ANALYSIS
  - Inverse probability weighting in Cox and logistic regression
  - Association between DNAm and future macrovascular events
  - Prediction of macrovascular events using methylation risk scores
  - CVD risk scores based on clinical risk factors
  - Polygenic risk score
  - Epigenetic clocks

SUPPLEMENTAL INFORMATION

Supplemental information can be found online at https://doi.org/10.1016/j.xcrm.2025.102290.

Received: January 23, 2025
Revised: May 28, 2025
Accepted: July 16, 2025
Published: August 7, 2025

REFERENCES

1. Fox, C.S., Coady, S., Sorlie, P.D., D'Agostino, R.B., Sr., Pencina, M.J., Vasan, R.S., Meigs, J.B., Levy, D., and Savage, P.J. (2007). Increasing cardiovascular disease burden due to diabetes mellitus: the Framingham Heart Study. Circulation *115*, 1544–1550. https://doi.org/10.1161/CIRCULATIONAHA.106.658948.
2. Gregg, E.W., Sattar, N., and Ali, M.K. (2016). The changing face of diabetes complications. Lancet Diabetes Endocrinol. *4*, 537–547. https://doi.org/10.1016/S2213-8587(16)30010-9.
3. Selvin, E., Lazo, M., Chen, Y., Shen, L., Rubin, J., McEvoy, J.W., Hoogeveen, R.C., Sharrett, A.R., Ballantyne, C.M., and Coresh, J. (2014). Diabetes mellitus, prediabetes, and incidence of subclinical myocardial damage. Circulation *130*, 1374–1382. https://doi.org/10.1161/CIRCULATIONAHA.114.010815.
4. Kelsey, M.D., Nelson, A.J., Green, J.B., Granger, C.B., Peterson, E.D., McGuire, D.K., and Pagidipati, N.J. (2022). Guidelines for Cardiovascular Risk Reduction in Patients With Type 2 Diabetes: JACC Guideline Comparison. J. Am. Coll. Cardiol. *79*, 1849–1857. https://doi.org/10.1016/j.jacc.2022.02.046.
5. Kavaric, N., Klisic, A., and Ninic, A. (2018). Cardiovascular Risk Estimated by UKPDS Risk Engine Algorithm in Diabetes. Open Med. *13*, 610–617. https://doi.org/10.1515/med-2018-0086.
6. D'Agostino, R.B., Sr., Vasan, R.S., Pencina, M.J., Wolf, P.A., Cobain, M., Massaro, J.M., and Kannel, W.B. (2008). General cardiovascular risk profile for use in primary care: the Framingham Heart Study. Circulation *117*, 743–753. https://doi.org/10.1161/CIRCULATIONAHA.107.699579.
7. Goff, D.C., Jr., Lloyd-Jones, D.M., Bennett, G., Coady, S., D'Agostino, R.B., Gibbons, R., Greenland, P., Lackland, D.T., Levy, D., O'Donnell, C.J., et al. (2014). 2013 ACC/AHA guideline on the assessment of cardiovascular risk: a report of the American College of Cardiology/American Heart Association Task Force on Practice Guidelines. Circulation *129*, S49–S73. https://doi.org/10.1161/01.cir.0000437741.48606.98.
8. McClelland, R.L., Jorgensen, N.W., Budoff, M., Blaha, M.J., Post, W.S., Kronmal, R.A., Bild, D.E., Shea, S., Liu, K., Watson, K.E., et al. (2015). 10-Year Coronary Heart Disease Risk Prediction Using Coronary Artery Calcium and Traditional Risk Factors: Derivation in the MESA (Multi-Ethnic Study of Atherosclerosis) With Validation in the HNR (Heinz Nixdorf Recall) Study and the DHS (Dallas Heart Study). J. Am. Coll. Cardiol. *66*, 1643–1653. https://doi.org/10.1016/j.jacc.2015.08.035.
9. SCORE2-Diabetes Working Group and the ESC Cardiovascular Risk Collaboration (2023). SCORE2-Diabetes: 10-year cardiovascular risk estimation in type 2 diabetes in Europe. Eur. Heart J. *44*, 2544–2556. https://doi.org/10.1093/eurheartj/ehad260.
10. van der Leeuw, J., van Dieren, S., Beulens, J.W.J., Boeing, H., Spijkerman, A.M.W., van der Graaf, Y., van der A, D.L., Nöthlings, U., Visseren, F.L.J., Rutten, G.E.H.M., et al. (2015). The validation of cardiovascular risk scores for patients with type 2 diabetes mellitus. Heart *101*, 222–229. https://doi.org/10.1136/heartjnl-2014-306068.
11. Dziopa, K., Asselbergs, F.W., Gratton, J., Chaturvedi, N., and Schmidt, A.F. (2022). Cardiovascular risk prediction in type 2 diabetes: a comparison of 22 risk scores in primary care settings. Diabetologia *65*, 644–656. https://doi.org/10.1007/s00125-021-05640-y.
12. Kengne, A.P., Patel, A., Colagiuri, S., Heller, S., Hamet, P., Marre, M., Pan, C.Y., Zoungas, S., Grobbee, D.E., Neal, B., et al. (2010). The Framingham and UK Prospective Diabetes Study (UKPDS) risk equations do not reliably estimate the probability of cardiovascular events in a large ethnically diverse sample of patients with diabetes: the Action in Diabetes and Vascular Disease: Preterax and Diamicron-MR Controlled Evaluation (ADVANCE) Study. Diabetologia *53*, 821–831. https://doi.org/10.1007/s00125-010-1681-4.
13. National Collaborating Centre for Chronic Conditions. Type 2 Diabetes: National Clinical Guideline for Management in Primary and Secondary Care (Update). London: Royal College of Physicians; 2008. p. 1-253. ISBN: 9781860163332. PMID: 21678628.
14. Fall, T., Gustafsson, S., Orho-Melander, M., and Ingelsson, E. (2018). Genome-wide association study of coronary artery disease among individuals with diabetes: the UK Biobank. Diabetologia *61*, 2174–2179. https://doi.org/10.1007/s00125-018-4686-z.
15. Qi, L., Parast, L., Cai, T., Powers, C., Gervino, E.V., Hauser, T.H., Hu, F.B., and Doria, A. (2011). Genetic susceptibility to coronary heart disease in type 2 diabetes: 3 independent studies. J. Am. Coll. Cardiol. *58*, 2675–2682. https://doi.org/10.1016/j.jacc.2011.08.054.
16. Agardh, E., Lundstig, A., Perfilyev, A., Volkov, P., Freiburghaus, T., Lindholm, E., Rönn, T., Agardh, C.D., and Ling, C. (2015). Genome-wide analysis of DNA methylation in subjects with type 1 diabetes identifies epigenetic modifications associated with proliferative diabetic retinopathy. BMC Med. *13*, 182. https://doi.org/10.1186/s12916-015-0421-5.
17. Reddy, M.A., Zhang, E., and Natarajan, R. (2015). Epigenetic mechanisms in diabetic complications and metabolic memory. Diabetologia *58*, 443–455. https://doi.org/10.1007/s00125-014-3462-y.
18. Schrader, S., Perfilyev, A., Ahlqvist, E., Groop, L., Vaag, A., Martinell, M., García-Calzón, S., and Ling, C. (2022). Novel Subgroups of Type 2 Diabetes Display Different Epigenetic Patterns That Associate With Future Diabetic Complications. Diabetes Care *45*, 1621–1630. https://doi.org/10.2337/dc21-2489.
19. Marchiori, M., Maguolo, A., Perfilyev, A., Maziarz, M., Martinell, M., Gomez, M.F., Ahlqvist, E., García-Calzón, S., and Ling, C. (2025). Blood-Based Epigenetic Biomarkers Associated With Incident Chronic Kidney Disease in Individuals With Type 2 Diabetes. Diabetes. *74*, 439–450. https://doi.org/10.2337/db24-0483.
20. Chen, Z., Miao, F., Paterson, A.D., Lachin, J.M., Zhang, L., Schones, D.E., Wu, X., Wang, J., Tompkins, J.D., Genuth, S., et al. (2016). Epigenomic profiling reveals an association between persistence of DNA methylation and metabolic memory in the DCCT/EDIC type 1 diabetes cohort. Proc. Natl. Acad. Sci. USA *113*, E3002–E3011. https://doi.org/10.1073/pnas.1603712113.
21. Fernandez-Sanles, A., Sayols-Baixeras, S., Curcio, S., Subirana, I., Marrugat, J., and Elosua, R. (2018). DNA Methylation and Age-Independent Cardiovascular Risk, an Epigenome-Wide Approach: The REGICOR Study (REgistre GIroni del COR). Arterioscler. Thromb. Vasc. Biol. *38*, 645–652. https://doi.org/10.1161/ATVBAHA.117.310340.
22. Agha, G., Mendelson, M.M., Ward-Caviness, C.K., Joehanes, R., Huan, T., Gondalia, R., Salfati, E., Brody, J.A., Fiorito, G., Bressler, J., et al. (2019). Blood Leukocyte DNA Methylation Predicts Risk of Future Myocardial Infarction and Coronary Heart Disease. Circulation *140*, 645–657. https://doi.org/10.1161/CIRCULATIONAHA.118.039357.
23. Reynolds, L.M., Taylor, J.R., Ding, J., Lohman, K., Johnson, C., Siscovick, D., Burke, G., Post, W., Shea, S., Jacobs, D.R., Jr., et al. (2014). Age-related variations in the methylome associated with gene expression in human monocytes and T cells. Nat. Commun. *5*, 5366. https://doi.org/10.1038/ncomms6366.
24. Sun, J., Singh, P., Shami, A., Kluza, E., Pan, M., Djordjevic, D., Michaelsen, N.B., Kennbäck, C., van der Wel, N.N., Orho-Melander, M., et al. (2023). Spatial Transcriptional Mapping Reveals Site-Specific Pathways Underlying Human Atherosclerotic Plaque Rupture. J. Am. Coll. Cardiol. *81*, 2213–2227. https://doi.org/10.1016/j.jacc.2023.04.008.
25. Zaina, S., Gonçalves, I., Carmona, F.J., Gomez, A., Heyn, H., Mollet, I.G., Moran, S., Varol, N., and Esteller, M. (2015). DNA methylation dynamics in human carotid plaques after cerebrovascular events. Arterioscler. Thromb. Vasc. Biol. *35*, 1835–1842. https://doi.org/10.1161/ATVBAHA.115.305630.
26. Zaina, S., Heyn, H., Carmona, F.J., Varol, N., Sayols, S., Condom, E., Ramírez-Ruz, J., Gomez, A., Gonçalves, I., Moran, S., and Esteller, M. (2014). DNA methylation map of human atherosclerosis. Circ. Cardiovasc. Genet. *7*, 692–700. https://doi.org/10.1161/CIRCGENETICS.113.000441.

CellPress
OPEN ACCESS

27. Andersen, A.M., Philibert, R.A., Gibbons, F.X., Simons, R.L., and Long, J. (2017). Accuracy and utility of an epigenetic biomarker for smoking in populations with varying rates of false self-report. Am. J. Med. Genet. B Neuropsychiatr. Genet. *174*, 641–650. https://doi.org/10.1002/ajmg.b.32555.

28. LaBarre, B.A., Goncearenco, A., Petrykowska, H.M., Jaratlerdsiri, W., Bornman, M.S.R., Hayes, V.M., and Elnitski, L. (2019). MethylToSNP: identifying SNPs in Illumina DNA methylation array data. Epigenetics Chromatin *12*, 79. https://doi.org/10.1186/s13072-019-0321-6.

29. Morieri, M.L., Gao, H., Pigeyre, M., Shah, H.S., Sjaarda, J., Mendonca, C., Hastings, T., Buranasupkajorn, P., Motsinger-Reif, A.A., Rotroff, D.M., et al. (2018). Genetic Tools for Coronary Risk Assessment in Type 2 Diabetes: A Cohort Study From the ACCORD Clinical Trial. Diabetes Care *41*, 2404–2413. https://doi.org/10.2337/dc18-0709.

30. Mansour Aly, D., Dwivedi, O.P., Prasad, R.B., Käräjämäki, A., Hjort, R., Thangam, M., Åkerlund, M., Mahajan, A., Udler, M.S., Florez, J.C., et al. (2021). Genome-wide association analyses highlight etiological differences underlying newly defined subtypes of diabetes. Nat. Genet. *53*, 1534–1542. https://doi.org/10.1038/s41588-021-00948-2.

31. Bernabeu, E., McCartney, D.L., Gadd, D.A., Hillary, R.F., Lu, A.T., Murphy, L., Wrobel, N., Campbell, A., Harris, S.E., Liewald, D., et al. (2023). Refining epigenetic prediction of chronological and biological age. Genome Med. *15*, 12. https://doi.org/10.1186/s13073-023-01161-y.

32. Horvath, S., Oshima, J., Martin, G.M., Lu, A.T., Quach, A., Cohen, H., Felton, S., Matsuyama, M., Lowe, D., Kabacik, S., et al. (2018). Epigenetic clock for skin and blood cells applied to Hutchinson Gilford Progeria Syndrome and ex vivo studies. Aging *10*, 1758–1775. https://doi.org/10.18632/aging.101508.

33. Levine, M.E., Lu, A.T., Quach, A., Chen, B.H., Assimes, T.L., Bandinelli, S., Hou, L., Baccarelli, A.A., Stewart, J.D., Li, Y., et al. (2018). An epigenetic biomarker of aging for lifespan and healthspan. Aging *10*, 573–591. https://doi.org/10.18632/aging.101414.

34. Zhang, Q., Vallerga, C.L., Walker, R.M., Lin, T., Henders, A.K., Montgomery, G.W., He, J., Fan, D., Fowdar, J., Kennedy, M., et al. (2019). Improved precision of epigenetic clock estimates across tissues and its implication for biological ageing. Genome Med. *11*, 54. https://doi.org/10.1186/s13073-019-0667-1.

35. Zhang, Y., Wilson, R., Heiss, J., Breitling, L.P., Saum, K.U., Schöttker, B., Holleczek, B., Waldenberger, M., Peters, A., and Brenner, H. (2017). DNA methylation signatures in peripheral blood strongly predict all-cause mortality. Nat. Commun. *8*, 14617. https://doi.org/10.1038/ncomms14617.

36. Youden, W.J. (1950). Index for rating diagnostic tests. Cancer *3*, 32–35. https://doi.org/10.1002/1097-0142(1950)3:1<32::aid-cncr2820030106>3.0.co;2-3.

37. Supek, F., Bošnjak, M., Škunca, N., and Šmuc, T. (2011). REVIGO summarizes and visualizes long lists of gene ontology terms. PLoS One *6*, e21800. https://doi.org/10.1371/journal.pone.0021800.

38. Ling, C., Bacos, K., and Rönn, T. (2022). Epigenetics of type 2 diabetes mellitus and weight change - a tool for precision medicine? Nat. Rev. Endocrinol. *18*, 433–448. https://doi.org/10.1038/s41574-022-00671-w.

39. Ronn, T., Ofori, J.K., Perfilyev, A., Hamilton, A., Pircs, K., Eichelmann, F., Garcia-Calzon, S., Karagiannopoulos, A., Stenlund, H., Wendt, A., et al. (2023). Genes with epigenetic alterations in human pancreatic islets impact mitochondrial function, insulin secretion, and type 2 diabetes. Nat. Commun. *14*, 8040. https://doi.org/10.1038/s41467-023-43719-9.

40. Yin, Y., Morgunova, E., Jolma, A., Kaasinen, E., Sahu, B., Khund-Sayeed, S., Das, P.K., Kivioja, T., Dave, K., Zhong, F., et al. (2017). Impact of cytosine methylation on DNA binding specificities of human transcription factors. Science *356*, eaaj2239. https://doi.org/10.1126/science.aaj2239.

41. Garcia-Calzon, S., Perfilyev, A., Martinell, M., Ustinova, M., Kalamajski, S., Franks, P.W., Bacos, K., Elbere, I., Pihlajamaki, J., Volkov, P., et al. (2020). Epigenetic markers associated with metformin response and intolerance in drug-naive patients with type 2 diabetes. Sci. Transl. Med. *12*, eaaz1803. https://doi.org/10.1126/scitranslmed.aaz1803.

42. Boeing, H., Korfmann, A., and Bergmann, M.M. (1999). Recruitment procedures of EPIC-Germany. European Investigation into Cancer and Nutrition. Ann. Nutr. Metab. *43*, 205–215. https://doi.org/10.1159/000012787.

43. Scirica, B.M., Bhatt, D.L., Braunwald, E., Raz, I., Cavender, M.A., Im, K., Mosenzon, O., Udell, J.A., Hirshberg, B., Pollack, P.S., et al. (2016). Prognostic Implications of Biomarker Assessments in Patients With Type 2 Diabetes at High Cardiovascular Risk: A Secondary Analysis of a Randomized Clinical Trial. JAMA Cardiol. *1*, 989–998. https://doi.org/10.1001/jamacardio.2016.3030.

44. Grant, P.J., and Cosentino, F. (2019). The 2019 ESC Guidelines on diabetes, pre-diabetes, and cardiovascular diseases developed in collaboration with the EASD: New features and the 'Ten Commandments' of the 2019 Guidelines are discussed by Professor Peter J. Grant and Professor Francesco Cosentino, the Task Force chairmen. Eur. Heart J. *40*, 3215–3217. https://doi.org/10.1093/eurheartj/ehz687.

45. Joseph, J.J., Deedwania, P., Acharya, T., Aguilar, D., Bhatt, D.L., Chyun, D.A., Di Palo, K.E., Golden, S.H., Sperling, L.S., et al.; American Heart Association Diabetes Committee of the Council on Lifestyle and Cardiometabolic Health; Council on Arteriosclerosis Thrombosis and Vascular Biology; Council on Clinical Cardiology; and Council on Hypertension (2022). Comprehensive Management of Cardiovascular Risk Factors for Adults With Type 2 Diabetes: A Scientific Statement From the American Heart Association. Circulation *145*, e722–e759. https://doi.org/10.1161/CIR.0000000000001040.

46. Price, A.H., Weir, C.J., Welsh, P., McLachlan, S., Strachan, M.W.J., Sattar, N., and Price, J.F. (2017). Comparison of non-traditional biomarkers, and combinations of biomarkers, for vascular risk prediction in people with type 2 diabetes: The Edinburgh Type 2 Diabetes Study. Atherosclerosis *264*, 67–73. https://doi.org/10.1016/j.atherosclerosis.2017.07.009.

47. van der Leeuw, J., Beulens, J.W.J., van Dieren, S., Schalkwijk, C.G., Glatz, J.F.C., Hofker, M.H., Verschuren, W.M.M., Boer, J.M.A., van der Graaf, Y., Visseren, F.L.J., et al. (2016). Novel Biomarkers to Improve the Prediction of Cardiovascular Event Risk in Type 2 Diabetes Mellitus. J. Am. Heart Assoc. *5*, e003048. https://doi.org/10.1161/JAHA.115.003048.

48. Fadini, G.P., Rigato, M., Cappellari, R., Bonora, B.M., and Avogaro, A. (2017). Long-term Prediction of Cardiovascular Outcomes by Circulating CD34+ and CD34+CD133+ Stem Cells in Patients With Type 2 Diabetes. Diabetes Care *40*, 125–131. https://doi.org/10.2337/dc16-1755.

49. Welsh, P., Rankin, N., Li, Q., Mark, P.B., Würtz, P., Ala-Korpela, M., Marre, M., Poulter, N., Hamet, P., Chalmers, J., et al. (2018). Circulating amino acids and the risk of macrovascular, microvascular and mortality outcomes in individuals with type 2 diabetes: results from the ADVANCE trial. Diabetologia *61*, 1581–1591. https://doi.org/10.1007/s00125-018-4619-x.

50. International Diabetes Federation. IDF Diabetes Atlas, 10th edn. Brussels, Belgium: 2021. https://www.diabetesatlas.org.

51. Chen, H.Y., Kuo, S., Su, P.F., Wu, J.S., and Ou, H.T. (2020). Health Care Costs Associated With Macrovascular, Microvascular, and Metabolic Complications of Type 2 Diabetes Across Time: Estimates From a Population-Based Cohort of More Than 0.8 Million Individuals With Up to 15 Years of Follow-up. Diabetes Care *43*, 1732–1740. https://doi.org/10.2337/dc20-0072.

52. Caro, J.J., Ward, A.J., and O'Brien, J.A. (2002). Lifetime costs of complications resulting from type 2 diabetes in the U.S. Diabetes Care *25*, 476–481. https://doi.org/10.2337/diacare.25.3.476.

53. Tobias, D.K., Merino, J., Ahmad, A., Aiken, C., Benham, J.L., Bodhini, D., Clark, A.L., Colclough, K., Corcoy, R., Cromer, S.J., et al. (2023). Second international consensus report on gaps and opportunities for the clinical translation of precision diabetes medicine. Nat. Med. *29*, 2438–2457. https://doi.org/10.1038/s41591-023-02502-5.

54. American Diabetes Association (2021). 10. Cardiovascular Disease and Risk Management: Standards of Medical Care in Diabetes-2021. Diabetes Care *44*, S125–S150. https://doi.org/10.2337/dc21-S010.

55. Ahmadloo, S., Ling, K.H., Fazli, A., Larijani, G., Ghodsian, N., Mohammadi, S., Amini, N., Hosseinpour Sarmadi, V., and Ismail, P. (2022). Signature pattern of gene expression and signaling pathway in premature diabetic patients uncover their correlation to early age coronary heart disease. Diabetol. Metab. Syndr. *14*, 107. https://doi.org/10.1186/s13098-022-00878-x.

56. Gonzalez-Navarro, H., Vinue, A., Vila-Caballer, M., Fortuno, A., Beloqui, O., Zalba, G., Burks, D., Diez, J., and Andres, V. (2008). Molecular mechanisms of atherosclerosis in metabolic syndrome: role of reduced IRS2-dependent signaling. Arterioscler. Thromb. Vasc. Biol. *28*, 2187–2194. https://doi.org/10.1161/ATVBAHA.108.175299.

57. Shen, Z., Bei, Y., Lin, H., Wei, T., Dai, Y., Hu, Y., Zhang, C., and Dai, H. (2023). The role of class IIa histone deacetylases in regulating endothelial function. Front. Physiol. *14*, 1091794. https://doi.org/10.3389/fphys.2023.1091794.

58. Kong, Q., Hao, Y., Li, X., Wang, X., Ji, B., and Wu, Y. (2018). HDAC4 in ischemic stroke: mechanisms and therapeutic potential. Clin. Epigenetics *10*, 117. https://doi.org/10.1186/s13148-018-0549-1.

59. Zhang, L., Wang, Y., Yu, F., Li, X., Gao, H., and Li, P. (2021). CircHIPK3 Plays Vital Roles in Cardiovascular Disease. Front. Cardiovasc. Med. *8*, 733248. https://doi.org/10.3389/fcvm.2021.733248.

60. Shan, K., Liu, C., Liu, B.H., Chen, X., Dong, R., Liu, X., Zhang, Y.Y., Liu, B., Zhang, S.J., Wang, J.J., et al. (2017). Circular Noncoding RNA HIPK3 Mediates Retinal Vascular Dysfunction in Diabetes Mellitus. Circulation *136*, 1629–1642. https://doi.org/10.1161/CIRCULATIONAHA.117.029004.

61. Wang, W., Zhang, S., Xu, L., Feng, Y., Wu, X., Zhang, M., Yu, Z., and Zhou, X. (2021). Involvement of circHIPK3 in the pathogenesis of diabetic cardiomyopathy in mice. Diabetologia *64*, 681–692. https://doi.org/10.1007/s00125-020-05353-8.

62. Messaoudi, S., He, Y., Gutsol, A., Wight, A., Hébert, R.L., Vilmundarson, R.O., Makrigiannis, A.P., Chalmers, J., Hamet, P., Tremblay, J., et al. (2015). Endothelial Gata5 transcription factor regulates blood pressure. Nat. Commun. *6*, 8835. https://doi.org/10.1038/ncomms9835.

63. Lai, C.H., Pandey, S., Day, C.H., Ho, T.J., Chen, R.J., Chang, R.L., Pai, P.Y., Padma, V.V., Kuo, W.W., and Huang, C.Y. (2019). beta-catenin/LEF1/IGF-IIR Signaling Axis Galvanizes the Angiotensin-II- induced Cardiac Hypertrophy. Int. J. Mol. Sci. *20*, 4288. https://doi.org/10.3390/ijms20174288.

64. Gallego-Fabrega, C., Cullell, N., Soriano-Tárraga, C., Carrera, C., Torres-Aguila, N.P., Muiño, E., Cárcel-Márquez, J., de Moura, M.C., Fernández-Sanlés, A., Esteller, M., et al. (2020). DNA methylation of MMPs and TIMPs in atherothrombosis process in carotid plaques and blood tissues. Oncotarget *11*, 905–912. https://doi.org/10.18632/oncotarget.27469.

65. Istas, G., Declerck, K., Pudenz, M., Szic, K.S.V., Lendinez-Tortajada, V., Leon-Latre, M., Heyninck, K., Haegeman, G., Casasnovas, J.A., Tellez-Plaza, M., et al. (2017). Identification of differentially methylated BRCA1 and CRISP2 DNA regions as blood surrogate markers for cardiovascular disease. Sci. Rep. *7*, 5120. https://doi.org/10.1038/s41598-017-03434-0.

66. Yin, Y., Xie, Z., Chen, D., Guo, H., Han, M., Zhu, Z., and Bi, J. (2022). Integrated investigation of DNA methylation, gene expression and immune cell population revealed immune cell infiltration associated with atherosclerotic plaque formation. BMC Med. Genomics *15*, 108. https://doi.org/10.1186/s12920-022-01259-z.

67. Wei, L., Zhao, S., Wang, G., Zhang, S., Luo, W., Qin, Z., Bi, X., Tan, Y., Meng, M., Qin, J., et al. (2018). SMAD7 methylation as a novel marker in atherosclerosis. Biochem. Biophys. Res. Commun. *496*, 700–705. https://doi.org/10.1016/j.bbrc.2018.01.121.

68. Bacos, K., Gillberg, L., Volkov, P., Olsson, A.H., Hansen, T., Pedersen, O., Gjesing, A.P., Eiberg, H., Tuomi, T., Almgren, P., et al. (2016). Blood-based biomarkers of age-associated epigenetic changes in human islets associate with insulin secretion and diabetes. Nat. Commun. *7*, 11089. https://doi.org/10.1038/ncomms11089.

69. Yoshida, T., Kato, K., Yokoi, K., Oguri, M., Watanabe, S., Metoki, N., Yoshida, H., Satoh, K., Aoyagi, Y., Nozawa, Y., and Yamada, Y. (2010). Association of genetic variants with hemorrhagic stroke in Japanese individuals. Int. J. Mol. Med. *25*, 649–656. https://doi.org/10.3892/ijmm_00000388.

70. Miller, R.G., Mychaleckyj, J.C., Onengut-Gumuscu, S., Feingold, E., Orchard, T.J., and Costacou, T. (2023). DNA methylation and 28-year cardiovascular disease risk in type 1 diabetes: the Epidemiology of Diabetes Complications (EDC) cohort study. Clin. Epigenetics *15*, 122. https://doi.org/10.1186/s13148-023-01539-0.

71. Chybowska, A.D., Gadd, D.A., Cheng, Y., Bernabeu, E., Campbell, A., Walker, R.M., McIntosh, A.M., Wrobel, N., Murphy, L., Welsh, P., et al. (2024). Epigenetic Contributions to Clinical Risk Prediction of Cardiovascular Disease. Circ. Genom. Precis. Med. *17*, e004265. https://doi.org/10.1161/CIRCGEN.123.004265.

72. He, Y., Chen, X., Liu, M., Zuo, L., Zhai, Z., Zhou, L., Li, G., Chen, L., Qi, G., Jing, C., and Hao, G. (2023). The potential DNA methylation markers of cardiovascular disease in patients with type 2 diabetes. BMC Med. Genomics *16*, 242. https://doi.org/10.1186/s12920-023-01689-3.

73. Rodriguez, J.D., Perez, A., and Lozano, J.A. (2010). Sensitivity analysis of kappa-fold cross validation in prediction error estimation. IEEE Trans. Pattern Anal. Mach. Intell. *32*, 569–575. https://doi.org/10.1109/TPAMI.2009.187.

74. Dayeh, T., Tuomi, T., Almgren, P., Perfilyev, A., Jansson, P.A., de Mello, V.D., Pihlajamäki, J., Vaag, A., Groop, L., Nilsson, E., and Ling, C. (2016). DNA methylation of loci within ABCG1 and PHOSPHO1 in blood DNA is associated with future type 2 diabetes risk. Epigenetics *11*, 482–488. https://doi.org/10.1080/15592294.2016.1178418.

75. Dayeh, T., Volkov, P., Salö, S., Hall, E., Nilsson, E., Olsson, A.H., Kirkpatrick, C.L., Wollheim, C.B., Eliasson, L., Rönn, T., et al. (2014). Genome-wide DNA methylation analysis of human pancreatic islets from type 2 diabetic and non-diabetic donors identifies candidate genes that influence insulin secretion. PLoS Genet. *10*, e1004160. https://doi.org/10.1371/journal.pgen.1004160.

76. Nilsson, E., Jansson, P.A., Perfilyev, A., Volkov, P., Pedersen, M., Svensson, M.K., Poulsen, P., Ribel-Madsen, R., Pedersen, N.L., Almgren, P., et al. (2014). Altered DNA methylation and differential expression of genes influencing metabolism and inflammation in adipose tissue from subjects with type 2 diabetes. Diabetes. *63*, 2962–2976. https://doi.org/10.2337/db13-1459.

77. Garcia-Calzon, S., Schrader, S., Perfilyev, A., Martinell, M., Ahlqvist, E., and Ling, C. (2023). DNA methylation partially mediates antidiabetic effects of metformin on HbA1c levels in individuals with type 2 diabetes. Diabetes Res. Clin. Pract. *202*, 110807. https://doi.org/10.1016/j.diabres.2023.110807.

78. Jonsson, J., Perfilyev, A., Kugelberg, U., Skog, S., Lindstrom, A., Ruhrmann, S., Ofori, J.K., Bacos, K., Ronn, T., Ost, A., and Ling, C. (2025). Impact of excess sugar on the whole genome DNA methylation pattern in human sperm. Epigenomics *17*, 89–104. https://doi.org/10.1080/17501911.2024.2439782.

79. Perfilyev, A., Dahlman, I., Gillberg, L., Rosqvist, F., Iggman, D., Volkov, P., Nilsson, E., Risérus, U., and Ling, C. (2017). Impact of polyunsaturated and saturated fat overfeeding on the DNA-methylation pattern in human adipose tissue: a randomized controlled trial. Am. J. Clin. Nutr. *105*, 991–1000. https://doi.org/10.3945/ajcn.116.143164.

80. Ronn, T., Volkov, P., Davegardh, C., Dayeh, T., Hall, E., Olsson, A.H., Nilsson, E., Tornberg, A., Dekker Nitert, M., Eriksson, K.F., et al. (2013). A six months exercise intervention influences the genome-wide DNA methylation pattern in human adipose tissue. PLoS Genet. *9*, e1003572. https://doi.org/10.1371/journal.pgen.1003572.

81. Oussalah, A., Rischer, S., Bensenane, M., Conroy, G., Filhine-Tresarrieu, P., Debard, R., Forest-Tramoy, D., Josse, T., Reinicke, D., Garcia, M., et al. (2018). Plasma mSEPT9: A Novel Circulating Cell-free DNA-Based Epigenetic Biomarker to Diagnose Hepatocellular Carcinoma. EBioMedicine *30*, 138–147. https://doi.org/10.1016/j.ebiom.2018.03.029.

82. Volkov, P., Bacos, K., Ofori, J.K., Esguerra, J.L.S., Eliasson, L., Rönn, T., and Ling, C. (2017). Whole-Genome Bisulfite Sequencing of Human Pancreatic Islets Reveals Novel Differentially Methylated Regions in Type 2 Diabetes Pathogenesis. Diabetes. *66*, 1074–1085. https://doi.org/10.2337/db16-0996.

83. de Winther, M.P.J., Bäck, M., Evans, P., Gomez, D., Goncalves, I., Jørgensen, H.F., Koenen, R.R., Lutgens, E., Norata, G.D., Osto, E., et al. (2023). Translational opportunities of single-cell biology in atherosclerosis. Eur. Heart J. *44*, 1216–1230. https://doi.org/10.1093/eurheartj/ehac686.

84. Du, P., Kibbe, W.A., and Lin, S.M. (2008). lumi: a pipeline for processing Illumina microarray. Bioinformatics *24*, 1547–1548. https://doi.org/10.1093/bioinformatics/btn224.

85. Johnson, W.E., Li, C., and Rabinovic, A. (2007). Adjusting batch effects in microarray expression data using empirical Bayes methods. Biostatistics *8*, 118–127. https://doi.org/10.1093/biostatistics/kxj037.

86. Teschendorff, A.E., Marabita, F., Lechner, M., Bartlett, T., Tegner, J., Gomez-Cabrero, D., and Beck, S. (2013). A beta-mixture quantile normalization method for correcting probe design bias in Illumina Infinium 450 k DNA methylation data. Bioinformatics *29*, 189–196. https://doi.org/10.1093/bioinformatics/bts680.

87. Phipson, B., Maksimovic, J., and Oshlack, A. (2016). missMethyl: an R package for analyzing data from Illumina's HumanMethylation450 platform. Bioinformatics *32*, 286–288. https://doi.org/10.1093/bioinformatics/btv560.

88. Patro, R., Duggal, G., Love, M.I., Irizarry, R.A., and Kingsford, C. (2017). Salmon provides fast and bias-aware quantification of transcript expression. Nat. Methods *14*, 417–419. https://doi.org/10.1038/nmeth.4197.

89. Soneson, C., Love, M.I., and Robinson, M.D. (2015). Differential analyses for RNA-seq: transcript-level estimates improve gene-level inferences. F1000Res. *4*, 1521. https://doi.org/10.12688/f1000research.7563.2.

90. Robinson, M.D., McCarthy, D.J., and Smyth, G.K. (2010). edgeR: a Bioconductor package for differential expression analysis of digital gene expression data. Bioinformatics *26*, 139–140. https://doi.org/10.1093/bioinformatics/btp616.

91. Hoffman, G.E., and Schadt, E.E. (2016). variancePartition: interpreting drivers of variation in complex gene expression studies. BMC Bioinf. *17*, 483. https://doi.org/10.1186/s12859-016-1323-z.

92. Min, J.L., Hemani, G., Davey Smith, G., Relton, C., and Suderman, M. (2018). Meffil: efficient normalization and analysis of very large DNA methylation datasets. Bioinformatics *34*, 3983–3989. https://doi.org/10.1093/bioinformatics/bty476.

93. Robin, X., Turck, N., Hainard, A., Tiberti, N., Lisacek, F., Sanchez, J.C., and Müller, M. (2011). pROC: an open-source package for R and S+ to analyze and compare ROC curves. BMC Bioinf. *12*, 77. https://doi.org/10.1186/1471-2105-12-77.

94. Kundu, S., Aulchenko, Y.S., van Duijn, C.M., and Janssens, A.C.J.W. (2011). PredictABEL: an R package for the assessment of risk prediction models. Eur. J. Epidemiol. *26*, 261–264. https://doi.org/10.1007/s10654-011-9567-4.

95. Buuren, S.v., and Groothuis-Oudshoorn, K. (2011). mice: Multivariate Imputation by Chained Equations inR. J. Stat. Softw. *45*, 1–67. https://doi.org/10.18637/jss.v045.i03.

96. Wang, Y., Grant, O.A., Zhai, X., McDonald-Maier, K.D., and Schalkwyk, L.C. (2024). Insights into ageing rates comparison across tissues from recalibrating cerebellum DNA methylation clock. Geroscience *46*, 39–56. https://doi.org/10.1007/s11357-023-00871-w.

97. Rovite, V., Wolff-Sagi, Y., Zaharenko, L., Nikitina-Zake, L., Grens, E., and Klovins, J. (2018). Genome Database of the Latvian Population (LGDB): Design, Goals, and Primary Results. J. Epidemiol. *28*, 353–360. https://doi.org/10.2188/jea.JE20170079.

98. Prentice, R.L. (1986). A case-cohort design for epidemiologic cohort studies and disease prevention trials. Biometrika *73*, 1–11. https://doi.org/10.1093/biomet/73.1.1.

99. McCartney, D.L., Walker, R.M., Morris, S.W., McIntosh, A.M., Porteous, D.J., and Evans, K.L. (2016). Identification of polymorphic and off-target probe binding sites on the Illumina Infinium MethylationEPIC BeadChip. Genom. Data *9*, 22–24. https://doi.org/10.1016/j.gdata.2016.05.012.

100. Seaman, S.R., and White, I.R. (2013). Review of inverse probability weighting for dealing with missing data. Stat. Methods Med. Res. *22*, 278–295. https://doi.org/10.1177/0962280210395740.

101. Uno, H., Cai, T., Tian, L., and Wei, L.J. (2007). Evaluating Prediction Rules for t-Year Survivors With Censored Regression Models. J. Am. Stat. Assoc. *102*, 527–537. https://doi.org/10.1198/016214507000000149.

102. Salas, L.A., Koestler, D.C., Butler, R.A., Hansen, H.M., Wiencke, J.K., Kelsey, K.T., and Christensen, B.C. (2018). An optimized library for reference-based deconvolution of whole-blood biospecimens assayed using the Illumina HumanMethylationEPIC BeadArray. Genome Biol. *19*, 64. https://doi.org/10.1186/s13059-018-1448-7.

103. Dawes, K., Andersen, A., Reimer, R., Mills, J.A., Hoffman, E., Long, J.D., Miller, S., and Philibert, R. (2021). The relationship of smoking to cg05575921 methylation in blood and saliva DNA samples from several studies. Sci. Rep. *11*, 21627. https://doi.org/10.1038/s41598-021-01088-7.

104. Gulati, M., Levy, P.D., Mukherjee, D., Amsterdam, E., Bhatt, D.L., Birtcher, K.K., Blankstein, R., Boyd, J., Bullock-Palmer, R.P., Conejo, T., et al. (2021). 2021 AHA/ACC/ASE/CHEST/SAEM/SCCT/SCMR Guideline for the Evaluation and Diagnosis of Chest Pain: A Report of the American College of Cardiology/American Heart Association Joint Committee on Clinical Practice Guidelines. Circulation *144*, e368–e454. https://doi.org/10.1161/CIR.0000000000001029.

105. Fransquet, P.D., Wrigglesworth, J., Woods, R.L., Ernst, M.E., and Ryan, J. (2019). The epigenetic clock as a predictor of disease and mortality risk: a systematic review and meta-analysis. Clin. Epigenetics *11*, 62. https://doi.org/10.1186/s13148-019-0656-7.

STAR★METHODS

KEY RESOURCES TABLE

REAGENT or RESOURCE	SOURCE	IDENTIFIER
**Biological samples**		
Human blood samples	This paper	N/A
Human plaques	Carotid Plaque Imaging Project (CPIP) biobank	ClinicalTrials.gov: NCT05821894
**Deposited data**		
Human blood DNA methylation datasets from ANDISand ANDIU	This paper	LUDC repository (https://www.ludc.lu.se/resources/ludc-repository): LUDC2023.12.1
DNA methylation datasets from human postmortem donor-matched atherosclerotic and nonatherosclerotic portions of aortas	Zaina et al.[26]	Gene Expression Omnibus (https://www.ncbi.nlm.nih.gov/geo/): GSE46401
DNA methylation datasets from asymptomatic (stable) or symptomatic carotid plaques collected after cerebrovascular events	Zaina et al.[25]	Gene Expression Omnibus (https://www.ncbi.nlm.nih.gov/geo/): GSE66500
RNA-seq dataset from human carotid plaques	Sun et al.[24]	Accessible upon reasonable request
Human blood DNA methylation datasets from OPTIMED	This paper	LUDC repository (https://www.ludc.lu.se/resources/ludc-repository): LUDC2023.12.1
Human blood DNA methylation datasets from EPIC-Postdam	This paper	https://www.dife.de/en/research/cooperations/epic-study/.
**Software and algorithms**		
R software	R Foundation for Statistical Computing	https://www.r-project.org/
Gentra Puregene Blood Kit	Qiagen	https://www.qiagen.com/us/products/discovery-and-translational-research/dna-rna-purification/dna-purification/genomic-dna/gentra-puregene-blood-kit
EZ DNA Methylation Kit	Zymo Research	https://www.zymoresearch.com/products/ez-dna-methylation-kit
Infinium MethylationEPIC BeadChip	Illumina Inc.	https://www.illumina.com/products/by-type/microarray-kits/infinium-methylation-epic.html
Methylumi (R package)	Bioconductor	https://bioconductor.org/packages/methylumi
lumi (R package)	Du et al.[84]	https://bioconductor.org/packages/lumi
COMBAT	Johnson et al.[85]	https://academic.oup.com/biostatistics/article/8/1/118/252073?login=true
BMIQ	Teschendorff et al.[86]	https://aeteschendorff-lab.github.io/software/BMIQ/
missMethyl (R package)	Phipson et al.[87]	https://bioconductor.org/packages/missMethyl
REVIGO	Supek et al.[37]	http://revigo.irb.hr/
Salmon	Patro et al.[88]	https://combine-lab.github.io/salmon/
Tximport (R package)	Soneson et al.[89]	https://bioconductor.org/packages/tximport
edgeR (R package)	Robinson et al.[90]	https://bioconductor.org/packages/edgeR
variancePartition (R package)	Hoffman and Schadt.[91]	https://bioconductor.org/packages/variancePartition
meffil (R package)	Min et al.[92]	https://github.com/perishky/meffil
pROC R package	Robin et al.[93]	https://cran.r-project.org/package=pROC
PredictABEL R package	Kundu et al.[94]	https://cran.r-project.org/package=PredictABEL
MethylToSNP tool	LaBarre B et al.[28]	https://github.com/elnitskilab/MethylToSNP
CVrisk R package	CRAN	https://cran.r-project.org/package=CVrisk
mice R package	Stef van Buuren & Karin Groothuis-Oudshoorn.[95]	https://cran.r-project.org/package=mice
dnaMethyAge R package	Wang et al.[96]	https://github.com/yiluyucheng/dnaMethyAge?tab=readme-ov-file

EXPERIMENTAL MODEL AND STUDY PARTICIPANT DETAILS

Discovery cohort: The prospective cohort for macrovascular events in T2D

The All New Diabetics in Scania (ANDIS) cohort is an ongoing study aiming to recruit all new cases of diabetes in Scania, Sweden.[18] Blood samples for DNA extraction and clinical variables are collected when registered with ANDIS, mainly corresponding to diagnosis of diabetes. ANDIS is linked with hospital clinical chemistry and regional health care databases allowing information from disease debut and prospective outcomes. Medication data is available through the national drug registry, registering data when patients pick up medication from the pharmacy. In this study, only participants diagnosed with T2D were considered.

The All New Diabetics in Uppsala County (ANDiU, http://www.andiu.se/) cohort includes newly diagnosed individuals with diabetes from the County of Uppsala, Sweden. After diabetes diagnosis, patients participating in ANDiU provided written informed consent and blood was collected. ANDiU is linked to the national diabetes and drug registry.

Figure 1 illustrates the study design. To test if epigenetic biomarkers could discriminate between individuals with T2D who will develop future macrovascular events or not, 752 newly diagnosed individuals with T2D from ANDIS and ANDiU with available DNAm data from blood taken at registration, and without any previous macrovascular events (n = 123 were excluded), were included in *the prospective cohort for macrovascular events in T2D*. Among 752 newly diagnosed individuals with T2D, 102 individuals developed macrovascular events, defined as myocardial infarction, angina, stroke, or ischemic heart disease, requiring hospitalization, within 7.2 years (Table 1, individuals with iME). Myocardial infarction, angina and ischemic heart disease were defined by International Classification of Diseases (ICD)-10 codes I20-I21, I24, I251 and I253-I259. Stroke was defined by ICD-10 codes I60-I61 and I63-I64. 650 newly diagnosed individuals with T2D were considered controls (macrovascular event censored individuals) since they did not develop macrovascular events during the follow-up period. Table 1 presents clinical characteristics of the 752 individuals included in *the prospective cohort for macrovascular events in T2D*. Individuals with known macrovascular events before registration were excluded.

The mean age of individuals who developed iMEs was 65 years (SD = 10.1) with 35% being female, whereas the mean age of the 650 individuals who did not develop any iMEs was 59.9 years (SD = 11.5), with 45% being female. This cohort was based predominantly on individuals of Northern European ancestry, although specific information on ethnicity was not available. ANDIS was approved by Lund's ethical review board (584/2006, 2011/354, 2014/198, 2016/529) while ANDiU was approved by Uppsala's ethical review board (2011/155). All participants provided written informed consent.

Validation cohorts

OPTIMED cohort

For validation, we used previously generated MethylationEPIC BeadChip data from blood of 21 newly diagnosed individuals with T2D from OPTIMED, free of macrovascular events at registration, of whom 10 developed macrovascular events within 11 years follow-up.[41] Figure S2A presents the selection of these 21 individuals. The mean age of individuals who developed iMEs was 56.8 years (SD = 11.6) with 70% being female, whereas the mean age of those who did not develop iMEs was 54.8 years (SD = 13.6) with 73% being female (Table S5A). This cohort comprised individuals from the Latvian population, although ethnicity is not available. OPTIMED is part of the Latvian National Research Program "BIOMEDICINE"[97] and was approved by Central Medical Ethics Committee of Latvia (No.01–29.1/22). Written informed consent was obtained for all the participants.

EPIC-Potsdam cohort

Blood cell DNAm was measured in a case-cohort study nested in the European Prospective Investigation into Cancer and Nutrition (EPIC)-Potsdam cohort study. The study sample comprised a representative subset of EPIC-Potsdam (n = 1,070) and all incident CVD cases identified between baseline recruitment (1994–1998) and final censoring on November 30, 2006 (n = 427). These 427 subjects who developed iMEs had a mean age of 56 years (SD = 7.5) and 63.5% were females, whereas the 1,070 who did not develop iMEs during ~12 years of follow-up had a mean age of 50.2 years (SD = 8.9) and 61.5% were females (Table S5B). Although ethnicity was not explicitly reported, the cohort primarily consisted of individuals from Germany, as part of the EPIC-Potsdam study. EPIC-Potsdam protocol was approved by the ethics committee of the Medical Society of the State of Brandenburg, Germany.[42] All participants provided a statement of written informed consent prior to enrollment.

The case-cohort design was accounted for by assigning weights as proposed by Prentice.[98] These weights are realized by counting survival time of participants of the random subcohort fully (cases and non-cases) and survival time of incident cases outside the subcohort only at the date of diagnosis. Age was the underlying time variable, with entry time as age at baseline and exit time as age at event or censoring. Details on recruitment and study procedures were reported elsewhere.[42] Incident CVD was defined as incidence of primary nonfatal and fatal myocardial infarction and stroke defined by ICD-10 codes I21, I63.0-I63.9, I61.0-I61.9, I60.0 to I60.9, and I64.0-I64.9. Incidence of CVD was captured by participants' self-reports or based on information from the death certificates, which were validated by contacting the treating physicians. Inquired information included ICD-10 code, date of occurrence, and further information on symptoms and diagnostic criteria. For myocardial infarction, diagnostic criteria included clinical symptoms, electrocardiograms, cardiac enzymes, and known coronary heart disease. For stroke, diagnosis was based on anamnesis, clinical symptoms, computed tomography/magnetic resonance imaging, angiogram, lumbar puncture, echocardiogram, Doppler, and electrocardiograms, plus imaging techniques if available. Participants with silent cardiovascular events that had not been documented within 28 days after occurrence were excluded as non-verifiable cases from all analyses. The median accrued follow-up time was 8.4 years (interquartile range, 7.6–9.2 years).

Blood samples were taken at baseline and buffy coat fractions immediately separated and stored in liquid nitrogen tanks at −196°C until DNA extraction. DNAm was measured using the Illumina EPIC 850K array and raw data were processed and normalized using the R package meffil.[92]

Carotid plaque imaging project (CPIP) biobank

To assess the biological relevance of methylation sites, data from aortic and carotid plaques obtained from the CPIP biobank were used. For aortic tissue, postmortem donor-matched atherosclerotic and nonatherosclerotic portions of aortas were included (n = 15; mean age 65.5 years, SD = 11.7; 33.3% females).[26] For carotid plaques with available DNAm, we included symptomatic patients (n = 19; mean age of 74.0 years, SD = 8.7; 21.1% females) and asymptomatic counterparts (n = 19; mean age of 67.9 years, SD = 5.4; 31.6% females).[25] For carotid plaques with RNA-sequencing available, we included symptomatic patients (n = 51; median age of 75 years, IQR = 69.5–80; 35.3% females) and asymptomatic patients (n = 27; median age of 69 years, IQR = 60.5–71; 29.6% females).[24] All plaque samples were obtained from the CPIP biobank (Region Skåne, Malmö, Sweden; ClinicalTrials.gov ID NCT05821894). Given the lack of reported ethnicity information, results should be interpreted in the context of a predominantly Northern European population. The CPIP study followed the Declaration of Helsinki and was approved by the Swedish ethical committee (472/2005, 2014/904, 2017/89, 2018/63, 27–2020/3.1, 60/2008, 2012/209). Written informed consent was provided by all participants.

The Multi-Ethnic Study of Atherosclerosis (MESA) cohort

To further explore the biological role, we also analyzed publicly available DNAm and gene expression data from the MESA cohort (n = 1,264; mean age 60 years, SD = 10; 51% females).[23] The cohort included 590 individuals of Caucasian ancestry, 402 Hispanic and 272 African American subjects. The study protocol was approved by the Institutional Review Boards at Johns Hopkins Medical Institutions, University of Minnesota, Columbia University Medical Center, and Wake Forest University Health Sciences. All participants signed informed consent.

METHOD DETAILS

Phenotypes measurements in the prospective cohort for macrovascular events in T2D

Age, weight, and height were registered at the date when blood was drawn. HbA1c, cholesterol, triglyceride levels, eGFR and urinary albumin/creatinine ratio were obtained from the clinical chemistry database. These variables were measured at baseline, and we considered the closest available value before blood for methylation analysis were taken. In less than 10 patients, some variables were not available, and we then considered data over 3 months after blood sampling for methylation analysis. Medication was extracted from the drug registry if subjects retrieved medicine from the pharmacy within 6 months before blood sampling for methylation analysis using the following ATC codes for A10-diabetes, C10-lipid-lowering and C02-C03 and C08-C09-antihypertensive medications.

DNAm profiling in the prospective cohort for macrovascular events in T2D

DNA was extracted from blood with Gentra Puregene Blood kit (Qiagen, Hilden, Germany). Next, 500–1000 ng of DNA was bisulfite treated by EZ DNA methylation kit (Zymo Research, Orange, CA, USA). DNAm was analyzed using Infinium MethylationEPIC BeadChip (850K array, Illumina, CA, USA), targeting 853,307 sites, at Lund University. Samples were randomized across chips. Bisulfite converted DNA was hybridized to BeadChips according to Infinium HD assay protocol. BeadChips images were scanned using iScan. Raw fluorescence intensities were extracted using Methylumi package (https://bioconductor.org/packages/methylumi). Probes with mean detection p-values>0.01, rs-probes, cross-reactive probes, polymorphic probes,[99] Y chromosome and non-CpG probes were filtered away, leaving 816,597 probes for statistical analyses. M-values were calculated as $M = \log_2 \frac{\max(M,0)+1}{\max(U,0)+1}$, where M and U are methylated and unmethylated channel intensities, respectively, and these were used for bioinformatic analyses. Quantile normalization and background correction were then done with lumi package (https://bioconductor.org/packages/lumi/).[84] BMIQ and COMBAT corrected for type 2 probes and batch effects, respectively.[85,86] For easier interpretation of methylation data, M-values were converted into beta-values when presenting data.

Gene ontology of methylated sites associated with incident macrovascular events

Gene ontology (GO) analysis using gometh function in the missMethyl R package[87] was performed to find enriched biological processes of differentially methylated sites associated with iMEs. Gometh function takes significant methylation sites, maps them to Entrez Gene IDs, and tests for GO term enrichment, considering number of sites per gene on the EPIC array and multi-gene annotated sites. We selected significant biological processes (p < 0.01) and REViGO removed redundant GO terms.[37]

DNAm in human plaques

We tested if any of the identified methylation sites associated with iMEs, also have a biological role in target tissues of the disease, e.g., atherosclerotic plaques. We used HumanMethylation450 BeadChip methylation data (450k covering 485,577 sites) available on GEO from two cohorts; i) postmortem donor-matched atherosclerotic and nonatherosclerotic portions of aortas (n = 15, GEO:

GSE46401)[26] and ii) carotid atherosclerotic plaques collected from asymptomatic patients with stenosis (*n* = 19) or patients suffering cerebrovascular events (*n* = 19, GEO: GSE66500).[25] Characteristics of plaques donors were previously described.[25,26] Data was extracted if methylation sites associated with iMEs were also covered by 450k array in the plaque cohorts. Paired-sample and independent-sample t-tests were used to test for differences in the aortic and carotid plaque cohorts, respectively. FDR analysis corrected for multiple testing (considering FDR<5%).

RNA-sequencing of human carotid plaques

RNA-seq was performed on RNA isolated from the most stenotic region of human carotid plaques (*n* = 78; 27 asymptomatic and 51 symptomatic) using Illumina HiSeq2000 and NextSeq 500/550 platforms, as previously described.[24] The carotid plaques used for RNA-seq are from different people than the once donating plaques for the DNAm analysis described above.

Briefly, Salmon[88] was used to conduct transcript-level quantification based on transcriptome release 27 of GENCODE in mapping-based mode. Tximport[89] was used to summarize gene counts and all gene counts were normalized between samples using a trimmed mean of M-values (TMM) by edgeR,[90] giving gene expressions as log2-transformed counts per million (CPM) after voom transformation. After that, differential gene expression (DEG) analysis comparing plaques from symptomatic and asymptomatic patients was performed using a linear mixed model, adjusting for age, gender, and diabetes as fixed effects, and accounting for sequencing platform as a random effect. P-value from F-test was reported. DEG analysis was implemented using the R package variancePartition.[91]

QUANTIFICATION AND STATISTICAL ANALYSIS

Statistical analyses were performed using R Software. To assess differences in clinical characteristics between individuals with iMEs and controls in *the prospective cohort for macrovascular events in T2D*, Mann-Whitney tests and χ^2 tests were used for continuous and categorical variables, respectively. *A priori* power calculation was performed, indicating a statistical power of 96% ($\alpha = 0.05$) was achieved to find 2% differences in methylation (SD = 0.05) between 650 controls and 102 iMEs in *the prospective cohort for macrovascular events in T2D* (Figure S4).

Inverse probability weighting in Cox and logistic regression

The sampling weights (Wsamp) used in Cox and logistic regression were calculated using inverse probability of sampling weights[100] to account for any sampling bias potentially introduced when sampling into our study. These weights were calculated as total number of controls or iMEs calculated in respective whole cohort (ANDIS or ANDiU) divided by the number of controls or iMEs available in *the prospective cohort for macrovascular events in T2D*.

In addition to sampling weights, censoring weights (Wcens) were used in logistic regression to account for potential bias introduced due to censoring (drop-out during follow-up due to reasons unrelated to the study). Wcens were calculated using inverse probability of censoring weighting[101] where the probability of being censored was estimated using Kaplan-Meier estimator with censoring treated as the event of interest. Intuitively, this approach distributes the contribution of each censored participant among the individuals remaining at risk at the time of censoring of that participant. The final weights to be included in all logistic regression models were calculated as the product of Wcens x Wsamp.

Association between DNAm and future macrovascular events

To evaluate whether DNAm in blood was associated with iMEs in individuals with T2D, several weighted Cox regression models adjusted for cell-type composition and other confounders were used in *the prospective cohort for macrovascular events in T2D* (Figure 2A). Model 1 was adjusted for age, gender, BMI, and HbA1c. Since whole blood contains multiple cells, Model 2 was further adjusted for cell composition using a reference-based approach.[102] This deconvolution technique allows to estimate relative proportions of blood cell types using blood-derived DNAm signatures of CD8T, CD4T, natural killer, B-cells, monocytes, and neutrophils, which were subsequently included as covariates in Model 2. To increase sensitivity of our study, 8 additional weighted Cox models were run adjusting for additional covariates (see Figure 2A). While all models were adjusted for age, gender, BMI, and HbA1c, models 3–10 were further adjusted for additional covariates including: lipid lowering and antihypertensive drugs, smoking based on a biomarker of smoking (methylation of *AHRR*, cg05575921) since smoking information was not available for most individuals and *AHRR* methylation is known to predict smoking with 98% accuracy,[103] HDL and LDL cholesterol levels, or triglyceride levels. Covariates include established risk factors for macrovascular events.[44,104] We then tested whether methylation sites associated with iMEs in Model 1 were also identified in Models 2–10. Model 1 was used to identify methylation sites (FDR <0.05) due to its balance between statistical power and adjustment for key covariates. Selected sites were further required to show *p*-values <0.05 across Models 2–10 to ensure robustness against potential clinical confounders. We also adjusted models by renal function, including eGFR and urinary albumin/creatinine ratio, and diabetes medication. At baseline, only one person had been diagnosed with retinopathy, and we did not adjust our models for this. HR are presented with 95% confidence intervals and associated *p*-values.

Weighted-Cox regression models adjusted for age, gender, BMI and HbA1c (i.e., Model 1) were run for validation analysis in OPTIMED and EPIC-Potsdam cohorts.

Prediction of macrovascular events using methylation risk scores

We generated a weighted MRS using selected methylation sites, i.e., sites associated with iMEs in weighted Cox regression models 1–10, and with ≥2% absolute differences in methylation between individuals with iMEs and controls (Figure 1). ≥2% is an arbitrary cut-off, which we used for selection of methylation sites that may be more robust and more likely to validate. MRS was calculated as sum of the methylation level at each selected site times the effect size for that site (beta coefficient from the weighted Cox model corresponding to the log(HR)[18] based on model 1).

To estimate predicted probability of iMEs using MRS, we used weighted logistic regression (weights = Wcens x Wsamp) using 5-fold cross-validation.[73] The data was split into 5 parts stratifying on gender and macrovascular event status. Iterating through the 5-folds, we used 80% of data for training and, remaining 20% for testing. We fitted weighted logistic models using data in the training set, with macrovascular events by the end of the follow-up as the outcome, the MRS as the main predictor, adjusting for clinical risk factors. The model was: macrovascular events ~ MRS + (age + gender + HbA1c + BMI + smoking (cg05575921 methylation) + diabetes medication + lipid-lowering medication + antihypertensives). The fitted model was then used to predict the probability of macrovascular events by the end of the follow-up time using the data in the test set. This procedure was repeated for each of the 5-folds. Predicted risks were obtained for each individual and quality of predictions were evaluated using ROC curves and C-statistics with macrovascular event as the outcome using pROC package for R.[93] The PredictABEL package[94] was used to calculate metrics of reclassification, including the categorical and continuous NRI and the IDI.

To test if methylation differences >2% between individuals with iMEs and controls may be explained by SNPs, we used MethylToSNP[28] to search for SNP-like methylation patterns.

CVD risk scores based on clinical risk factors

The SCORE2-Diabetes,[9] UKPDS,[5] Framingham,[6] ASCVD,[7] and MESA 10-year ASCVD[8] risk scores were calculated using CVrisk package (https://CRAN.R-project.org/package=CVrisk) including clinical risk factors for CVD in *the prospective cohort for macrovascular events in T2D*. These calculated risk scores were then tested for prediction of macrovascular events in the same cohort by calculating AUCs of ROC curves using cross-validation (k = 5) as it is explained in the "Prediction of macrovascular events using methylation risk scores" section of the STAR Methods. Methylation cut-off value of 68% on cg05575921 (*AHRR*) was used to discriminate smokers from nonsmokers, since AUC of cg05575921 methylation for predicting smoking was 0.98.[103] Systolic blood pressure measurements were available for 648 individuals (Table 1) and missing values were imputed (*mice* R package).[95]

Polygenic risk score

A PRS, including 204 SNPs associated with CAD in T2D,[29] was calculated for 461 individuals with available GWAS data in *the prospective cohort for macrovascular events in T2D* using previously described methods.[30]

Epigenetic clocks

Epigenetic clocks were included in the analysis as benchmarks of biological aging, given their established predictive value for morbidity and mortality, including some cardiovascular outcomes.[105] The dnaMethyAge R package[96] was used to calculate several epigenetic aging/clocks; Bernabeu_cAge_2023,[31] Horvath_Age_2018,[32] Levine_PhenoAge_2018,[33] ZhangQ_Age_2019[34] and ZhangY_Mortality Risk_2017,[35] using methylation data from *the prospective cohort for macrovascular events in T2D*. Bernabeu's, Horvath's and, ZhangQ's clocks predict chronological age. The other epigenetic clocks aim to estimate biological age as indicator of health span, potentially reflecting mortality risk. Mean values were imputed for missing methylation values. Table S7 presents number of methylation sites for each clock. Epigenetic clocks were then tested for prediction of macrovascular events in *the prospective cohort for macrovascular events in T2D* by calculating AUCs of ROC curves using cross-validation (k = 5), as explained in the "Prediction of macrovascular events using methylation risk scores" section of the STAR Methods.

Cell Reports Medicine, Volume *6*

Supplemental information

Epigenetic biomarkers predict macrovascular events in individuals with type 2 diabetes

Sonia García-Calzón, Alice Maguolo, Fabian Eichelmann, Andreas Edsfeldt, Alexander Perfilyev, Marlena Maziarz, Axel Lindström, Jiangming Sun, Monta Briviba, Matthias B. Schulze, Janis Klovins, Emma Ahlqvist, Isabel Gonçalves, and Charlotte Ling

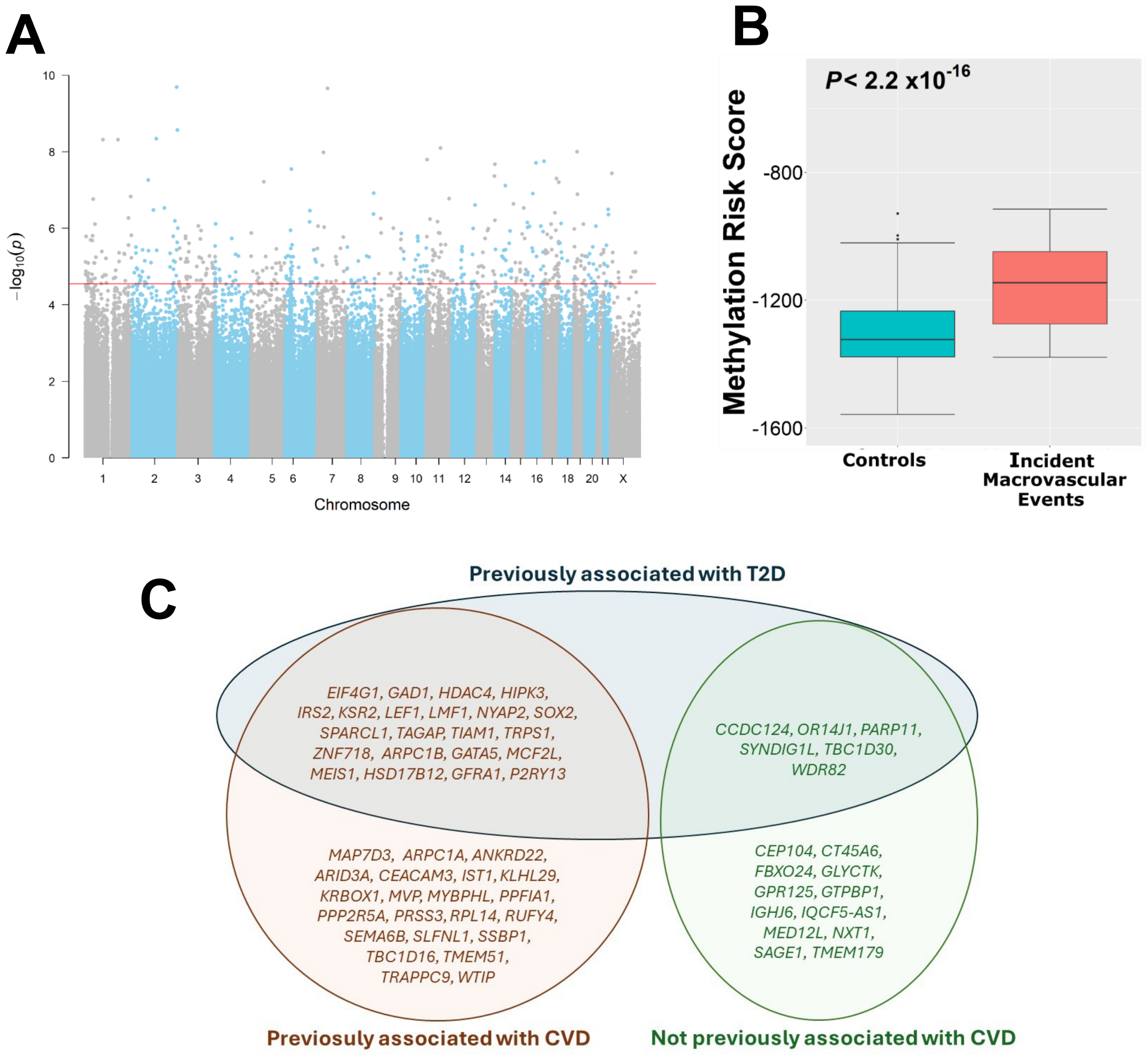

Figure S1. Blood DNA methylation is associated with incident macrovascular events (iME) in newly-diagnosed individuals with type 2 diabetes (T2D) and shows functional relevance in annotated genes, Related to Figures 2 and 6 (A) Manhattan plot showing the chromosomal distribution and significance levels of all sites analyzed (Illumina MethylationEPIC Beadchip array, 853,307 sites). The significance cut off (red line) was false discovery rate <5%. 461 sites were found significant(q<0.05) in the *prospective cohort for macrovascular events in T2D,* after adjusting for age, gender, BMI and HbA1c. **(B)** A Methylation Risk Score (MRS), based on the 87 methylation sites presented in Table S2B, was different between individuals who developed macrovascular events and controls who did not develop macrovascular events after 7 years of follow-up in newly-diagnosed individuals with T2D. The boxplot shows significantly different MRS between controls (n=650) and incident macrovascular events (n=102) in the *prospective cohort for macrovascular events in T2D* (*P* for Mann-Whitney U test $< 2.2 \times 10^{-16}$). **(C)** Venn Diagram of the 64 genes annotated to the 87 methylation sites associated with iME in individuals with T2D included in the MRS showing an overview of the biological relevance assessment performed based on systematic PubMed and GWAS Catalog searches. The Venn diagram shows the genes that has been previously associated with cardiovascular phenotypes in the PubMed search "gene AND Vascular Disease OR Cardiovascular Disease", "gene AND Myocardial Infarction", "gene AND Stroke", "gene AND Ischemic Heart Disease", "gene AND Angina" (also shown in Figure 6A) OR with CVD-related traits based on GWAS Catalog search (also shown in Figure 6B) and the genes that have been previously associated with diabetes in PubMed search "gene" AND "diabetes" or GWAS Catalog search of "diabetes" trait. Overall, 46 of 64 genes (72%) have been previously associated with CVD and 18 (28%) have not; however, 6 of these 18 genes were associated with diabetes.

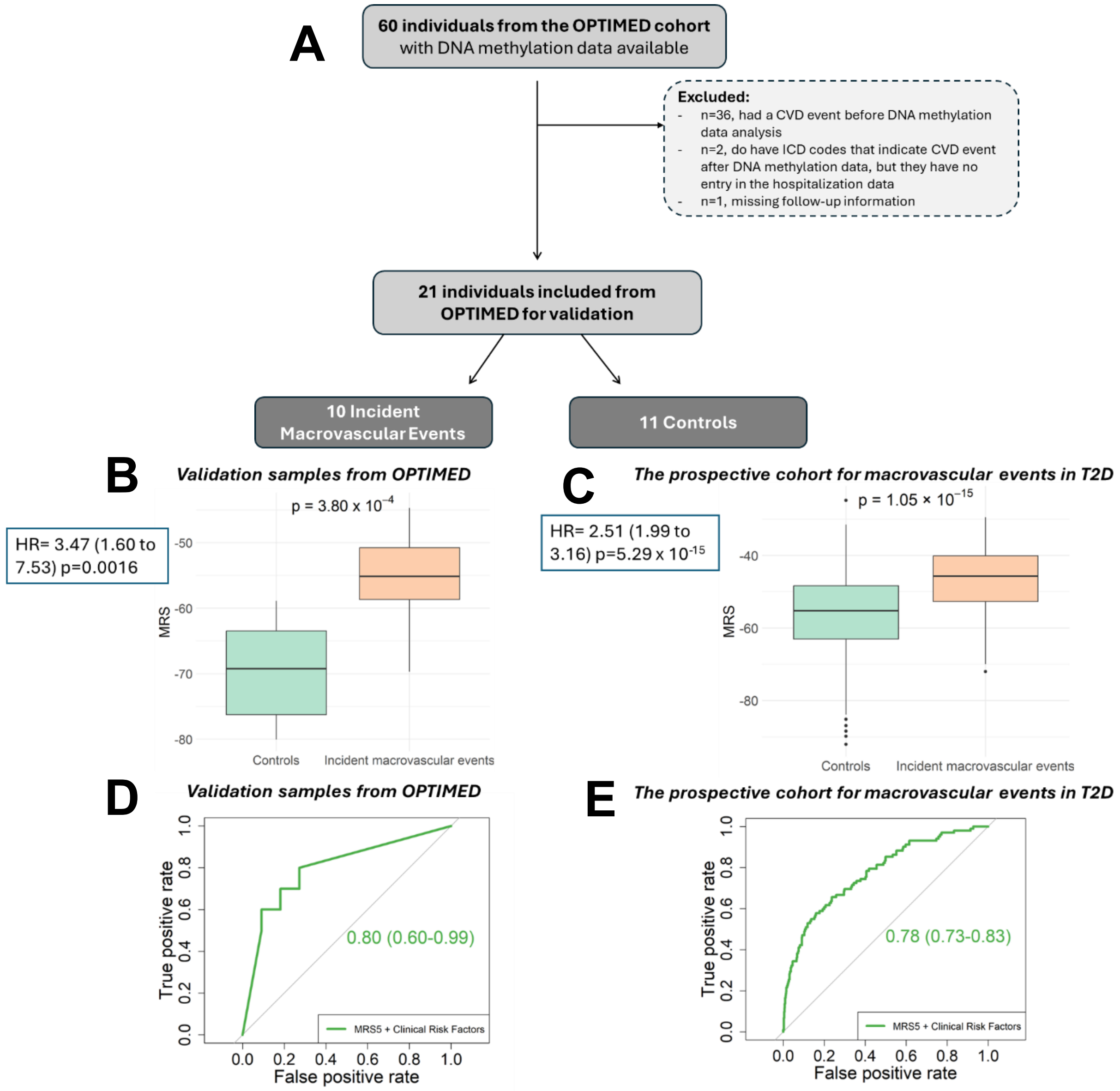

Figure S2. Validation of epigenetic markers associated with incident macrovascular events (iME) in newly-diagnosed individuals with type 2 diabetes (T2D) from the OPTIMED cohort. Related to Figure 1 and STAR methods. (A) Flowchart illustrating the selection and exclusion process of individuals from the OPTIMED cohort for validation, leading to the final analysis groups of iME and controls. **(B-E)** Methylation Risk Score (MRS_{5sites}), based on the five methylation sites presented in Table S5A, was different in blood taken from newly-diagnosed individuals with type 2 diabetes (T2D) between those who developed macrovascular events (iME) and controls who did not develop macrovascular events in (**B**) OPTIMED after 11 years of follow-up, and (**C**) *the prospective cohort for macrovascular events in T2D* after 7 years of follow-up. The boxplots show significantly different MRS_{5sites} between controls (n=11 and n=650, respectively) and iME (n=10 and n=102, respectively) in (**B**) OPTIMED (*P* for Mann-Whitney U test = =3.8x10^{-4}), and (**C**) *the prospective cohort for macrovascular events in T2D* (*P* for Mann-Whitney U test = 1.05x10^{-15}). Individuals who developed macrovascular events showed a 2.5- to 3.5-fold increased risk compared to controls per 1 SD increase in the MRS_{5sites}, with a hazard ratio (HR) of 3.47 (p=0.016) in OPTIMED, and an HR of 2.51 (p=5.29x10^{-15}) in *the prospective cohort for macrovascular events in T2D*. The MRS_{5sites} predicts macrovascular events in newly-diagnosed individuals with T2D in (**C**) OPTIMED after 11 years of follow-up using cross-validation, and (**D**) *the prospective cohort for macrovascular events in T2D* after 7 years of follow-up using cross-validation. ROC curves were generated with macrovascular events as the outcome and the predicted risks of each individual obtained using cross-validation (k=3 and 5, respectively) for the MRS_{5sites}, for the clinical risk factors (age + gender + HbA1c + BMI + smoking (cg05575921 methylation) + diabetes medication + lipid-lowering medication + antihypertensives) and for the combination of both the MRS and clinical risk factors, separately.

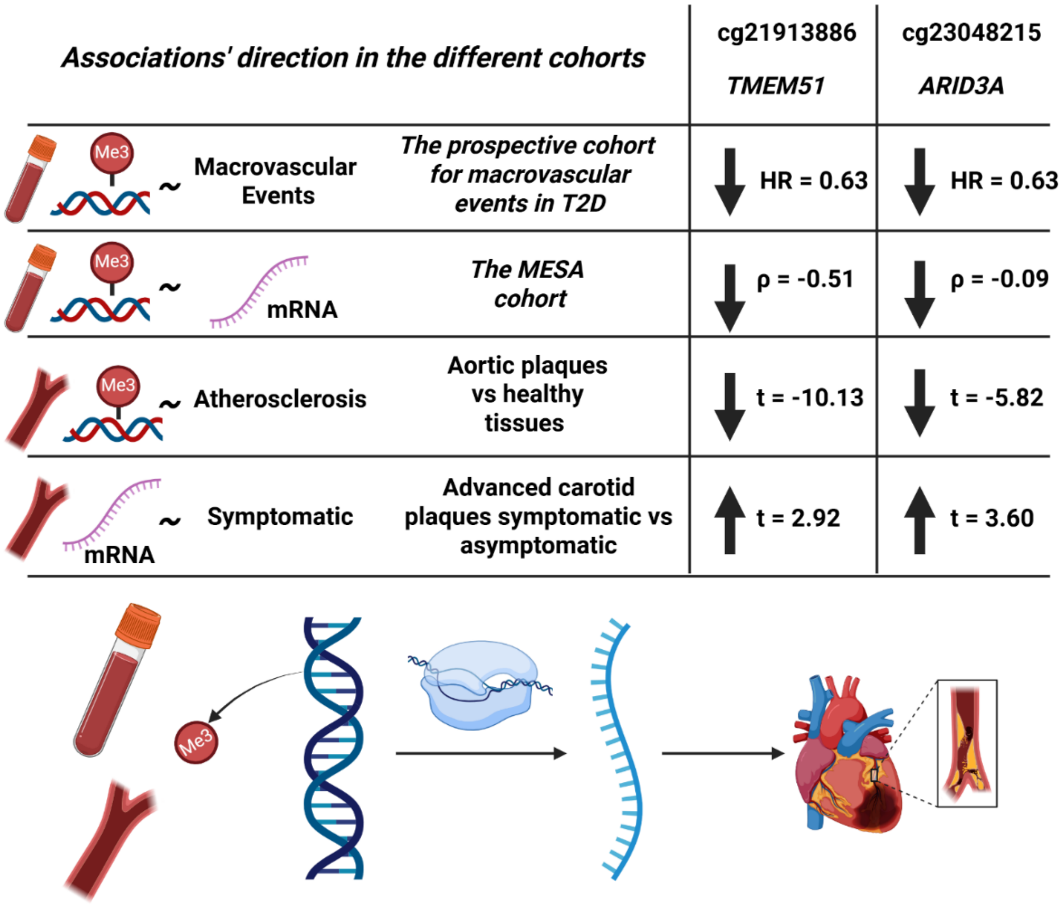

Figure S3. Concordance of associations' direction of DNA methylation and gene expression of the cg21913886 and cg23048215, respectively annotated to *TMEM51* and *ARID3A*, across different analyses and cohorts used in our study, **Related to Figure 6**. The figure shows that reduced DNA methylation in blood at these two CpG sites is associated with higher risk of macrovascular events in the *prospective cohort for macrovascular events in T2D* as well as with higher gene expression in the MESA cohort. Accordingly, we found that aortic plaques have reduced methylation at these sites compared to healthy aortic tissues and that symptomatic patients with advanced carotid plaques have higher expression of these genes compared to asymptomatic patients. Together, these analyses show that reduced methylation at cg21913886 and cg23048215 is associated with increased expression of *TMEM51* and *ARID3A*, respectively, that are associated with increased cardiovascular risk.

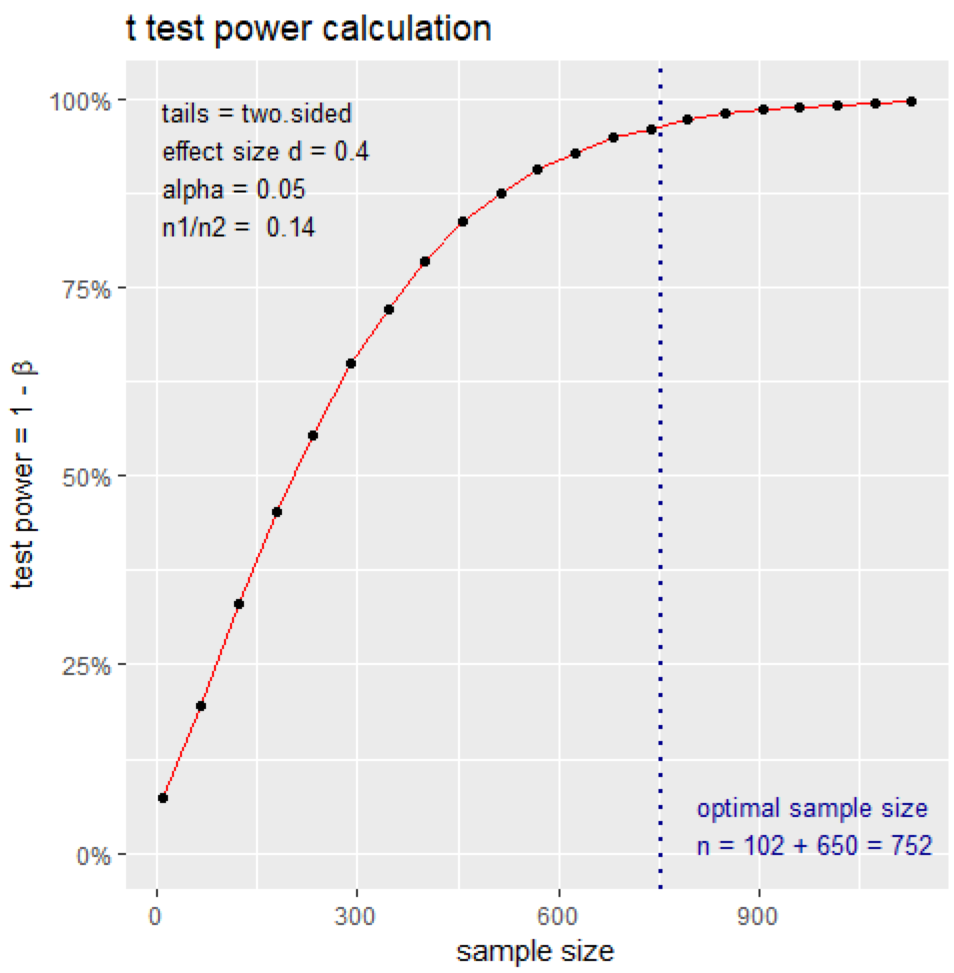

Figure S4. Statistical power calculations for the sample size in the *prospective cohort for macrovascular events in T2D*, Related to STAR Methods. A statistical power of 96% (α=0.05) was achieved to find 2% differences in methylation (SD=0.05) between controls (n=650) and incident macrovascular events (n=102) in the *prospective cohort for macrovascular events in T2D*. The pwr package in R was used to perform this plot.
